# The beneficial effects of ethanolic extract of *Sargassum serratifolium* in DNCB-induced mouse model of atopic dermatitis

**DOI:** 10.1038/s41598-024-62828-z

**Published:** 2024-06-05

**Authors:** Myeong-Jin Kim, Heeyeon Ryu, Hyeon Hak Jeong, Ji Yun Van, Ji Young Hwang, Ah-reum Kim, Jaeseong Seo, Kyoung Mi Moon, Won-Kyo Jung, Bonggi Lee

**Affiliations:** 1 Department of Food Science and Nutrition, Pukyong National University, 599-1, Daeyeondong, Nam-gu, Busan, 48513 Republic of Korea; 2 Department of Smart Green Technology Engineering, Pukyong National University, 599-1, Daeyeondong, Nam-gu, Busan, 48513 Republic of Korea; 3 Division of Biomedical Engineering and Research Center for Marine Integrated Bionics Technology, Pukyong National University, Busan, Republic of Korea; 4 Marine Integrated Biomedical Technology Center, The National Key Research Institutes, Pukyong National University, Busan, 48513 Republic of Korea

**Keywords:** Atopic dermatitis, Inflammation, *Sargassum serratifolium*, Macrophage, Keratinocyte, Hairless mice, Cell biology, Molecular biology, Biomarkers, Diseases, Medical research, Molecular medicine

## Abstract

Atopic dermatitis is a chronic complex inflammatory skin disorder that requires sustainable treatment methods due to the limited efficacy of conventional therapies. *Sargassum serratifolium*, an algal species with diverse bioactive substances, is investigated in this study for its potential benefits as a therapeutic agent for atopic dermatitis. RNA sequencing of LPS-stimulated macrophages treated with ethanolic extract of *Sargassum serratifolium* (ESS) revealed its ability to inhibit a broad range of inflammation-related signaling, which was proven in RAW 264.7 and HaCaT cells. In DNCB-induced BALB/c or HR-1 mice, ESS treatment improved symptoms of atopic dermatitis within the skin, along with histological improvements such as reduced epidermal thickness and infiltration of mast cells. ESS showed a tendency to improve serum IgE levels and inflammation-related cytokine changes, while also improving the mRNA expression levels of *Chi3l3, Ccr1, and Fcεr1a* genes in the skin. Additionally, ESS compounds (sargachromanol (SCM), sargaquinoic acid (SQA), and sargahydroquinoic acid (SHQA)) mitigated inflammatory responses in LPS-treated RAW264.7 macrophages. In summary, ESS has an anti-inflammatory effect and improves atopic dermatitis, ESS may be applied as a therapeutics for atopic dermatitis.

## Introduction

Atopic dermatitis, commonly known as eczema, is a chronic and relapsing inflammatory skin disorder that affects millions of individuals worldwide. This multifaceted condition is characterized by intense pruritus, erythema, dryness, and the formation of eczematous lesions^1^. Atopic dermatitis imposes a significant burden on both patients and healthcare systems, requiring comprehensive management strategies to alleviate symptoms, reduce inflammation, and improve the overall quality of life for those affected^2^.

Over the years, the management of atopic dermatitis has encompassed a variety of approaches, including topical corticosteroids, immunosuppressants, moisturizers, and lifestyle modifications. In mild cases, management is often effectively achieved with topical steroids. However, in cases of persistent symptoms or severity, a combination of local and systemic treatments is essential, which may include short-term systemic steroids or oral cyclosporine. Secondary options such as azathioprine, methotrexate, mycophenolate mofetil, antigen-specific immunotherapy, phototherapy, and biological agents like dupilumab are considered if standard treatment fails^3^. However, the long-term use of topical steroids can lead to side effects such as skin atrophy, telangiectasia, hypopigmentation, and cataracts, while biological agents, despite their efficacy, face significant challenges in widespread adoption due to high costs and limited accessibility^4^. Furthermore, the heterogeneity of the disease and varied patient responses to treatment emphasizes the importance of exploring alternative and natural therapies for managing atopic dermatitis. Therefore, the potential side effects and limited efficacy of conventional therapies have spurred interest in exploring alternative treatment options derived from natural sources^1,2^. In recent times, the utilization of marine-derived compounds has gained attention due to their rich bioactive components and potential therapeutic properties^5–7^.

*Sargassum*, a type of brown macroalgae, encompasses nearly 400 species^8^. However, only 78 of these species have been studied to understand their functional and phytochemical characteristics, with the majority of research focusing on 18 specific species^9^. The underutilization of *Sargassum* has resulted in marine pollution, which poses significant ecological, environmental, and economic concerns^10,11^. Notably, *Sargassum* blooms on ocean surfaces, and the accumulation of large mats of *Sargassum* on beaches can have detrimental effects on the health of local and visiting populations, fishing activities, and tourism^12–14^. Moreover, the removal of these mats presents challenges due to cost-effective issues^9^.

Despite the potential issues associated with *Sargassum*, it is worth noting that this genus has been traditionally consumed as a medicinal food, believed to protect against various diseases. For instance, studies have indicated that the consumption of brown seaweed, including *Sargassum* as a staple diet in Eastern Asian lifestyles, is correlated with a reduced incidence of cancers^15,16^. Additionally, other research has highlighted the antioxidant, anti-melanogenic, anti-inflammatory, anti-viral, anti-fungal, anti-bacterial, anti-bone loss, anti-metabolic syndrome, and neuroprotective activities associated with *Sargassum* consumption^9,17–21^. Consequently, exploring the development of *Sargassum* as a functional food or therapeutic agent holds promise in addressing marine pollution problems.

The anti-inflammatory potential of *Sargassum serratifolium* has been of particular interest, as chronic inflammation plays a pivotal role in the pathogenesis of atopic dermatitis. Inflammatory mediators such as cytokines, chemokines, and reactive oxygen species are known to contribute to disease progression and exacerbate symptoms. Studies have demonstrated that the bioactive components present in *Sargassum serratifolium* possess the ability to suppress the release of pro-inflammatory cytokines, inhibit the activation of immune cells, and scavenge reactive oxygen species, thus alleviating inflammation^22,23^. Furthermore, previous studies have demonstrated the potential of major compounds found in *Sargassum serratifolium* to inhibit skin aging^21,24^. However, until now, the impact of *Sargassum serratifolium* on atopic dermatitis in vivo has remained unknown.

In this research paper, we aim to critically evaluate the beneficial effects of *Sargassum serratifolium* in the management of atopic dermatitis using various mouse and cell models. Atopic dermatitis is characterized by dysregulated immune responses involving the activation of keratinocytes and subsequent inflammation^25–27^. Keratinocytes, which comprise the majority of epidermal cells, become activated in inflammatory states, leading to excessive proliferation and cytokine release, ultimately contributing to the onset of atopic dermatitis^27^. Furthermore, the macrophages and inflammatory cytokines expressed by T cells exacerbate the skin inflammation of atopic dermatitis^25^. Therefore, we conducted experiments to assess the anti-inflammatory effects of ESS using HaCaT keratinocyte cells and RAW 264.7 macrophage cells. Based on this, we conducted experiments to improve atopic dermatitis using HR-1 and BALB/c mice, widely recognized as suitable animal models for atopic dermatitis research, as they resemble human skin^28–30^. The induction of atopic-like dermatitis in these mice involved the application of 2,4-dinitrochlorobenzene (DNCB), a commonly used sensitizer. Subsequently, the ethanolic extract of *Sargassum serratifolium* (ESS) was topically applied to evaluate its therapeutic efficacy.

## Material and methods

### Reagents

Dimethyl sulfoxide (DMSO), lipopolysaccharide (LPS, E. Coli O111:B4), Phosphoric acid, Sulfanilamide, N-(1-Naphthyl)ethylenediamine dihydrochloride (NED), Recombinant Murine TNF-α, 1-chloro-2,4-dinitrobenzene (DNCB) was purchased from Sigma-Aldrich (MO, USA). All reagents related to cell culture were purchased from Welgene (Gyeongsangbuk-do, South Korea). Isoflurane (BKPharm, Gyeonggi-do, South Korea), Sodium Dodecyl Sulfate (Dongin Biotech, Seoul, South Korea), Propylene glycol (Samchun Chemicals, Seoul, South Korea), Ethanol (DAEJUNG, Gyeonggi-do, South Korea), 10% Neutral buffered formalin (Biosesang, Gyeonggi-do, South Korea), Acetone (JUNSEI, Tokyo, Japan), Olive Oil (Duksan, Seoul, South Korea). ESS samples were prepared based on a previous publication^31^ and dissolved in DMSO at various concentrations. all experimental protocols were approved by a named institutional and/or licensing committee/s.

### RNA purification and sequencing

We isolated total RNA from RAW 264.7 cells using the RiboEXTM reagent (GeneAll, Seoul, South Korea). To ensure the integrity of RNA, we utilized an Agilent 2200 TapeStation (Agilent Technologies, CA, USA), and only samples with a RIN value of 7 or higher were chosen for constructing the RNA library. For library preparation, we followed the manufacturer’s guidelines and employed the Illumina TruSeq Stranded Total RNA Library Prep Gold Kit (Illumina, CA, USA, #20020599). The initial step involved the removal of ribosomal RNA from total RNA using the Ribo-Zero Gold rRNA Removal Kit (Human/Mouse/Rat) (Illumina, CA, USA). Subsequently, we synthesized first- and second-strand cDNAs from the purified RNA samples, followed by adapter ligation to create the cDNA library. We quantified the cDNA library using the KAPA Library Quantification Kit for the Illumina Sequencing platform, following the qPCR Quantification Protocol Guide (KAPA BIOSYSTEMS, #KK4854). Additionally, we assessed the library quality using the TapeStation D1000 ScreenTape (Agilent Technologies, #5067-5582). For the sequencing process, we used the Illumina NovaSeq6000 (Illumina, Inc., CA, USA) with a paired-end configuration (2 × 151 bp).

### RNA-seq analysis

Sequencing was performed on an Illumina NovaSeq6000 (Illumina, Inc., San Diego, CA, USA), and mapping to the reference genome and read counting were performed using HISAT (version 2.1.0) and stringTie (2.1.3b)^32,33^.

### Differentially expressed gene analysis and protein network analysis

TMM normalization was performed using the EdgeR package (version 3.40) and the No Replicate method was utilized as described in “edgeR: Differential Analysis of Sequence Read Count Data User’s Guide” (square root variance was 0.1). Differentially expressed genes were screened based on the condition |logFC|> = 1 and FDR < 0.05. The filtered differentially expressed genes utilized KEGG analysis and STRING: Functional Protein Association Network (https://string-db.org) database.

### Cell culture

RAW 264.7 macrophage cell line was purchased from the Korean Cell Line Bank (Seoul, South Korea), and the Human keratinocyte cell line HaCaT was a kind gift from Korea Institute of Oriental Medicine (Daegu, South Korea). Each cell type was cultured in DMEM (Dulbecco’s modified Eagle’s medium) supplemented with 10% Fetal Bovine Serum (FBS) and 1% penicillin–streptomycin (P/S) at 37 °C in a humidified atmosphere containing 5% CO2.

### Cell viability

Cell viability was measured using an MTS assay (CellTiter96® AQueous One Solution Cell Proliferation Assay Kit, Promega, Madison, WI, USA). Briefly, RAW 264.7 cells (3 × 10^4^ cells/well) and HaCaT cells (0.8 × 10^4^ cells/well) were seeded in a 96-well plate. After 24 h, when the cells reached 80% confluency, they were treated with various concentrations of ESS diluted in serum-free DMEM (SFM) for 24 h. After 24 h of incubation, 5 μL of MTS reagent was added to each well and incubated at 37 °C for 1 h to develop the color. The absorbance was measured at 490 nm using a microplate reader. To test cell viability after inflammatory stimulation of RAW 264.7 and HaCaT cells, the same conditions were followed using 96-well plate seeding and pretreatment with concentrations of ESS diluted in SFM. After 1 h, RAW 264.7 and HaCaT cells were treated with lipopolysaccharide (LPS) (500 ng/mL) and TNF-α (20 ng/mL) to induce inflammation. After 24 h, 5 μL of MTS reagent was added to each well and incubated at 37 °C for 1 h to develop the color. The absorbance was measured at 490 nm using a microplate reader.

### Nitric Oxide (NO) assay

RAW 264.7 macrophage cell lines were seeded at 3 × 10^4^ cells/well in a 96-well plate. After 24 h, when 80% confluency was reached, the cells were pretreated with 2.5, 5 ppm concentrations of ESS diluted in SFM. After 1 h, the cells were treated with LPS (500 ng/mL) for 24 h. 100μL of the supernatant was taken and reacted for 30 min with 100μL of Griess reagent (8.5% Phosphoric acid, 1% Sulfanilamide, 0.1% naphthylethylenediamine dihydrochloride in deionized distilled water). The absorbance was measured at 540 nm using a microplate reader.

### Animals

In this experiment, female BALB/c mice (5 weeks old) were supplied by SAMTAKO (Gyeonggi-do, South Korea) and acclimatized in the animal laboratory for 3 weeks before being used in the experiments at 8 weeks of age. Female HOS:HR-1 mice (6 weeks old) were also supplied by Central Lab. Animal (Seoul, South Korea) and acclimatized in the animal laboratory for 1 week before being used in the experiments. During the adaptation and experiment periods, food and water were supplied ad libitum, and the temperature was maintained at 25 ± 2℃, humidity at 50 ± 5%, and a 12-h light/dark cycle. Animal testing procedures were performed in compliance with the regulations issued by the Animal Experimentation Ethics Committee of Pukyong National University (approval number: PKNUIACUC-2022–65, PKNUIACUC-2022–21). All methods are reported by ARRIVE guidelines (https://arriveguidelines.org) for the reporting of animal experiments.

### DNCB-induced BALB/c mouse atopic dermatitis model

**Figure 1:**
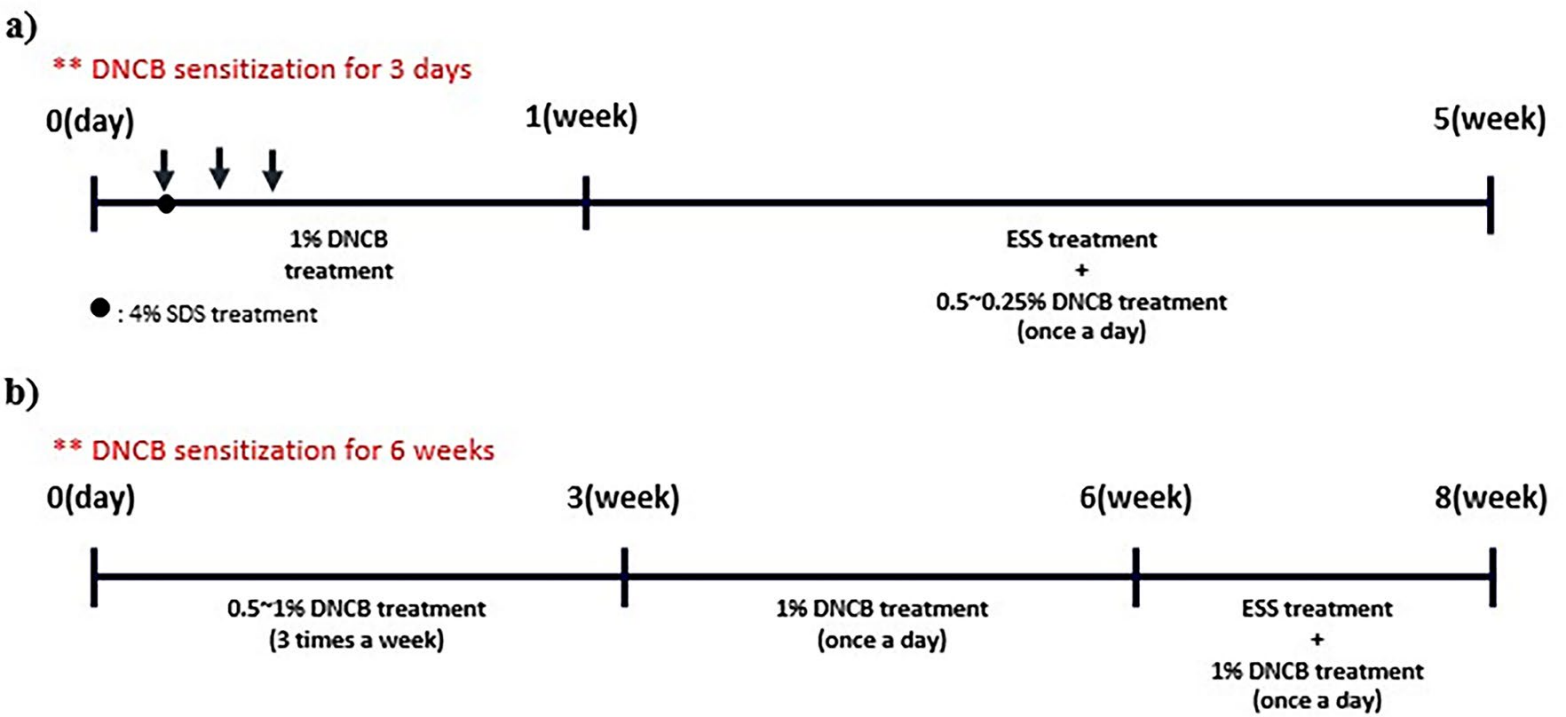
Experimental design of atopic dermatitis mouse models. (**a**) Experimental process of atopic dermatitis-like symptoms induction in BALB/c mice. (**b**) Long-term atopic dermatitis-like symptoms induction process in HR-1 mice.

BALB/c mice acclimatized in the laboratory for 3 weeks were induced with atopic dermatitis using DNCB. First, the mice were anesthetized, and their dorsal skin was thoroughly depilated using a depilatory and depilatory cream. During 24 h, a recovery period was provided for micro-wounds. Then, 200 μL of 4% SDS was applied to destroy the skin barrier. After 2 h, the mice were treated with a 1% DNCB 200 μL solution (acetone: olive oil = 3:1) for three days to induce atopic dermatitis-like symptoms^34,35^. Four days later, after confirming the induction of atopic dermatitis-like symptoms, 200–400 μL of ESS dissolved in solution (Propylene glycol: ethanol = 7:3) and 200 μL of DNCB (0.5–0.25%) solution were applied daily for 4 weeks to prevent natural healing (Fig. 1a)^34–36^. The experiment groups consisted of non-induced atopic dermatitis-like symptoms group (CON, n = 7), DNCB-induced atopic dermatitis-like symptoms group (DNCB, n = 7), DNCB + ESS 500 ppm group (ESS 500 ppm, n = 6), DNCB + ESS 1000 ppm group (ESS 1000 ppm, n = 6), DNCB + ESS 2000 ppm group (ESS 2000 ppm, n = 7), DNCB + Dexamethasone 2000 ppm group (DEX 2000 ppm, positive control, n = 5). At the end of the experiment, isoflurane was used to euthanize the mice. Right after the sacrifice, blood was collected from the heart and dorsal skin was collected. Blood was centrifuged at 3000 rpm and 20 min, and serum was stored at − 20 °C until the experiment. The dorsal skin of the mice was thoroughly depilated and rapidly frozen in liquid nitrogen and stored at − 70 °C.

#### Dermatitis score measurement

The severity of atopic dermatitis-like symptoms was evaluated every week after ESS treatment in BALB/c mice whose atopic dermatitis-like symptoms were induced by DNCB treatment. It was based on four pathological signs including erythema/hemorrhage, edema, excoriation/erosion, and scaling/dryness^36–38^. The scoring criteria were 0 (absent), 1 (mild), 2 (moderate), and 3 (severe) points according to severity, and the total clinical score was determined by the sum of all individual scores.

#### Histological analysis

To investigate the effect of ESS on histological changes in the skin of DNCB-induced atopic dermatitis-like symptoms in BALB/c mice and HR-1 mice, the dorsal skin was stained with Hematoxylin and Eosin (H&E) and Toluidine blue. The mouse dorsal skin fixed in 10% paraformaldehyde was embedded in paraffin and sectioned. For histological analysis, a commercially available company was used (Kyungpook National University Hospital). The sections were stained with H&E and toluidine blue, and histopathological changes were observed under a microscope.

#### HR-1 mouse atopic dermatitis model

Based on the experiments with BALB/c mice, we conducted a study using HR-1 mice, which have the most similar skin to human skin, to confirm the efficacy of ESS when atopic dermatitis-like symptoms are induced for a longer period using DNCB^28^. HR-1 mice, adapted to the laboratory for a week, were treated with 0.5–1% DNCB 200 μL three times a week for three weeks, and then with 1% DNCB 200 μL daily for the next three weeks to induce atopic dermatitis-like symptoms^39,40^. After confirming the induction of atopic dermatitis-like symptoms, 200–400 μL of ESS dissolved in solution (Propylene glycol: ethanol = 7:3) and 200 μL of DNCB (0.5–0.25%) solution were applied daily for four weeks to prevent natural healing (Fig. 1b). The experiment groups consisted of non-induced atopic dermatitis-like symptoms group (CON), DNCB-induced atopic dermatitis-like symptoms group (DNCB), DNCB + ESS 100 ppm group (ESS 100 ppm), DNCB + ESS 500 ppm group (ESS 500 ppm), with seven mice allocated per group. The other procedures including sacrifice and tissue collection are the same as those in the BALB/c mice.

#### Serum IgE and cytokine measurement

Serum IgE levels were measured using the LBIS Mouse IgE ELISA kit (FUJIFILM Wako Shibayagi Corporation, Gunma, Japan) according to the manufacturer’s instructions. Briefly, antibody-coated plates were incubated with standards and serum for 2 h. After washing, biotinylated anti-mouse IgE antibodies were incubated for 2 h. After washing, HRP-conjugated streptavidin was incubated for 1 h. Finally, after adding Chromogen (TMB) and reacting for 20 min, a stop solution was added and absorbance was measured at 450 nm using a microplate reader. All experiments were performed at 20–25 °C. Cytokine levels were analyzed using the Proteome Profiler Mouse XL Cytokine Array Kit (R&D, MN, USA) according to the manufacturer’s instructions. Briefly, membranes were incubated for 1 h at room temperature with a block buffer. After washing, the membrane was incubated with serum overnight at a temperature in the range of 2–8 °C. After washing, the membrane is incubated for 1 h at room temperature with a detection antibody cocktail. After washing again, the membrane was incubated with Streptavidin-HRP for 30 min at room temperature. Finally, the membrane was reacted with Chemi Reagent Mix for 1 min and exposed to X-ray film for 1–10 min. The resulting images were analyzed using Quick Spots Tool software (R&D, MN, USA) for quantification. Expression levels of cytokines were reported as signal intensities normalized to baseline on the same membrane.

#### Real-time polymerase chain reaction (RT-PCR)

RNA was isolated from cells and mouse dorsal skin tissue employing the RiboEX™ reagent (GeneAll, Seoul, South Korea) per the manufacturer’s guidelines. A homogenizer was used for RNA isolation from mouse dorsal skin. The extracted RNA was transcribed into cDNA using the SmartGene compact cDNA Synthesis kit (SMART GENE, Daejeon, South Korea). Real-time PCR was conducted utilizing the TOPreal™ SYBR Green qPCR PreMIX (Enzynomics, Daejeon, South Korea) on a QuantStudio™ 1 Real-Time PCR apparatus (Applied Biosystems, MA, USA). Target gene expression levels were adjusted to the reference genecorrection : β-actin; the fold variation was computed using the 2-ΔΔCT technique (Tables 1 and 2).

**Table 1:** The mouse primer sequences used in RT-PCR.

Gene	Primer sequences	
Olr1	Forward	CTT CCA TGG GCC CTT TAG C
	Reverse	AAC TGG CCA CCC AAA GAT TG
Trp73	Forward	CTT GCT TCT TGG GTG TAG GTT GT
	Reverse	TGT AGT CAA CCT CAG GCT TTG TG
Orm1	Forward	GCG CTG CAC ACG GTT CTT A
	Reverse	TGG GTT CTG AGC TTC CAA CAT
Chi3l3	Forward	TCT GGT GAA GGA AAT GCG TAA A
	Reverse	GCA GCC TTG GAA TGT CTT TCT C
Ccl20	Forward	CAA CTC CTG GAG CTG AGA ATG G
	Reverse	CCA TGC CAA AGC AAG GAA GA
Elane	Forward	CAG GAG CGC ACT CGA CAG A
	Reverse	GGG TCA AAG CCA TTC TCG AA
Itgb21	Forward	ACC ATG CTT CCT CCA CCC TAT
	Reverse	TTG TTG TGC TGC TGC TTC TGT
Anxa1	Forward	GGT CCT GGG TCA GCA GTG A
	Reverse	GCA GCA ACATCC GAG GAT ACA
Orm2	Forward	TCC TGC CGC TGT TGG AA
	Reverse	GGT CGC CTA TGG TGA TGT TGA
Alox5	Forward	CCC AGA GGA GCA TTT CAT TGA
	Reverse	TTC TTG CGG AAT CGG ATC A
Ccr1	Forward	TTT GTG GGT GAA CGG TTC TG
	Reverse	TGG TAT AGC CAC ATG CCT TTG A
Fcεr1a	Forward	TGG CTG CTC CTT CAG ACA TCT
	Reverse	GCA TCT GAT GTC AAA GGA TCC A

**Table 2:** The human primer sequences used in RT-PCR.

Gene	Primer sequences	
IL-6	Forward	AGG GCT CTT CGG GAA ATG T
	Reverse	GAA GAA GGA ATG CCC ATT AAC AAC
COX-2	Forward	AAG CAG GCT AAT ACT GAT AGG
	Reverse	TGT TGA AAA GTA GTT CTG GG
TNF-α	Forward	CTA TCT GGG AGG GGT CTT CC
	Reverse	ATG TTC GTC CTC CTC ACA GG

#### Statistical analysis

Experiment outcomes were denoted as the mean ± standard error of the mean (SEM) and relied upon data from a minimum of three separate trials. Differences between groups were assessed using GraphPad Prism 5.0 software (GraphPad Software, CA, USA) through a t-test or one-way analysis of variance (ANOVA) followed by Tukey’s multiple comparisons test. Specific statistics were mentioned in the figure legends.

## Results

### Anti-inflammatory effect of ESS in LPS-stimulated Raw 264.7 cells according to RNA sequencing analysis

**Figure 2:**
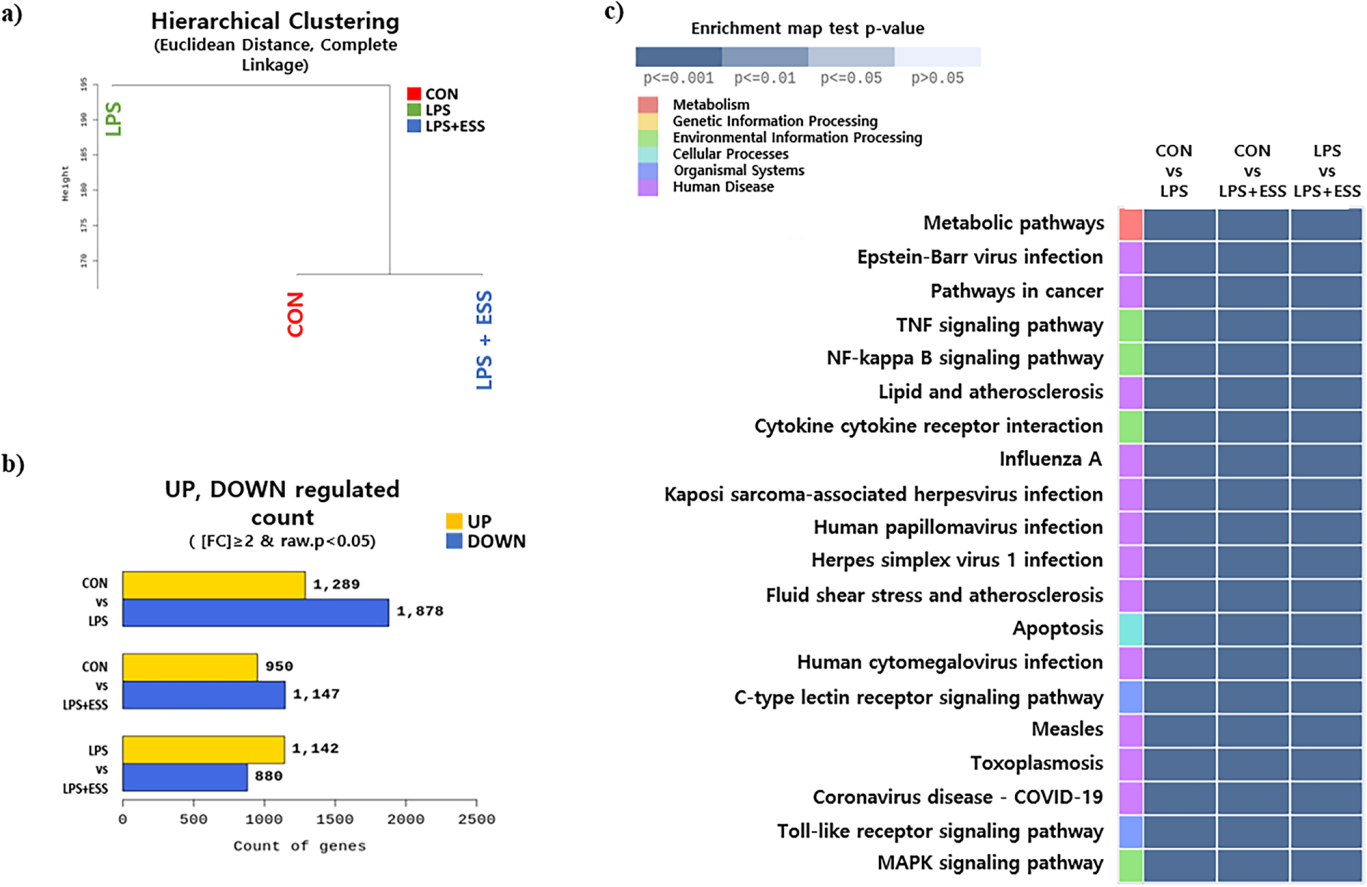
Analysis of gene expression modulation by ESS in LPS-treated RAW 264.7 cells using RNA sequencing. RNA sequencing analysis of RAW 264.7 cells treated with/without LPS or LPS + ESS. (**a**) Hierarchical clustering analysis for each sample and (**b**) the number of significant genes for each group is indicated based on fold change and *p*-value. (**c**) The heat map shows the KEGG pathway analysis result of RNA sequencing treated with ESS on LPS-treated macrophages. Among altered pathways of the LPS + ESS group vs. the LPS group, cell signaling pathways with significantly different gene expression (*p*-value < 0.001) between groups were selectively shown.

**Figure 3:**
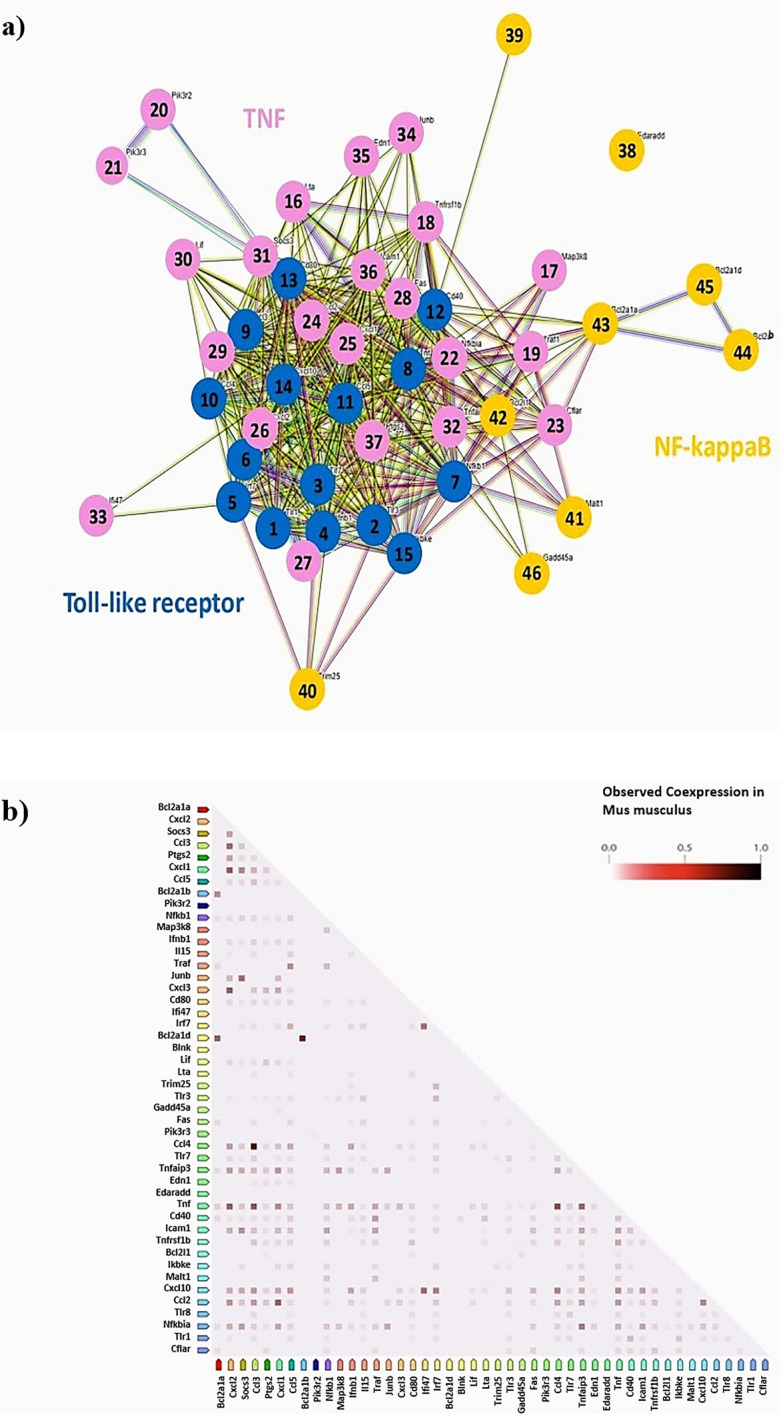
Protein network analysis of representative inflammatory signaling-related genes downregulated by ESS. We performed protein network analysis using genes associated with the Toll-like receptor signaling pathway, TNF signaling pathway, and NF-kappa B signaling pathway that were downregulated by ESS. (**a**) Toll-like receptor signaling pathway (genes 1 to 15: Tlr1, Tlr3, Tlr7, Ifnb1, Irf7, Tlr8, Nfkb1, Tnf, Ccl3, Ccl4, Ccl5, Cd40, Cd80, Cxcl10, Ikbke), TNF signaling pathway (genes 16 to 37: Lta, Map3k8, Tnfrsf1b, Traf1, Pik3r2, Pik3r3, Nfkbia, Cflar, Ccl2, Cxcl1, Cxcl2, Cxcl3, Fas, Il15, Lif, Socs3, Tnfaip3, Ifi47, Junb, Edn1, Icam1, Ptgs2), NF-kappa B signaling pathway (genes 38 to 46: Edaradd, Blnk, Trim25, Malt1, Bcl2l1, Bcl2a1a, Bcl2a1b, Bcl2a1d, Gadd45a). (**b**) Analysis of the correlation between genes associated with co-expression.

Activation of macrophages in the skin can lead to the development of chronic inflammatory diseases^25^. In this study, we investigated the effects of LPS-induced inflammatory responses on Raw 264.7 macrophages. Initially, we conducted RNA sequencing on Raw 264.7 cells that were treated with ESS following the induction of an inflammatory response by LPS. Using normalized values for each sample, we grouped samples based on the similarity in expression levels. As a result, the CON and LPS + ESS groups were grouped, distinct from the LPS group (Fig. 2a), implying that the CON and LPS + ESS groups exhibit similarity. The analysis of differentially expressed genes (FC ≥ 2, *p*-value < 0.05) revealed alterations in 2022 genes in the LPS vs. LPS + ESS groups, with an increase in expression observed in 1142 genes and a decrease in 880 genes (Fig. 2b). These results suggest that ESS treatment affects the regulation of genes involved in LPS-induced inflammatory responses. To analyze the inflammatory response regulation process in ESS, we performed a KEGG enrichment analysis based on the differentially expressed genes in the LPS + ESS group. The top 20 pathways ranked by *p*-values were visualized as a heatmap (Fig. 2c). Here, we focused on representative inflammatory response signaling pathways (tumor necrosis factor (TNF), nuclear factor (NF)-kappa B, Toll-like receptor) and conducted a protein–protein interaction analysis of the genetic information derived from KEGG enrichment analysis using the STRING database. The analysis revealed a network comprising 46 nodes with a notable count of 412 edges, significantly higher than the expected number of around 58 edges (PPI enrichment *p*-value < 1.0e-16) (Fig. 3a). Further co-expression analysis indicated that pairs like Tnf-C–C motif chemokine ligand (Ccl)4, Tnf-Ccl3, Ccl4-Ccl3, B-cell leukemia/lymphoma 2 related protein A1 (Bcl2a1)b-Bcl2a1d, C-X-C motif chemokine ligand (Cxcl)2-Cxcl1, and Cxcl1-Ccl2 had co-expression scores above 0.6, denoting significant interaction between proteins (Fig. 3b). These comprehensive results show that ESS treatment has the potential to modulate various markers related to inflammatory response.

**Figure 4:**
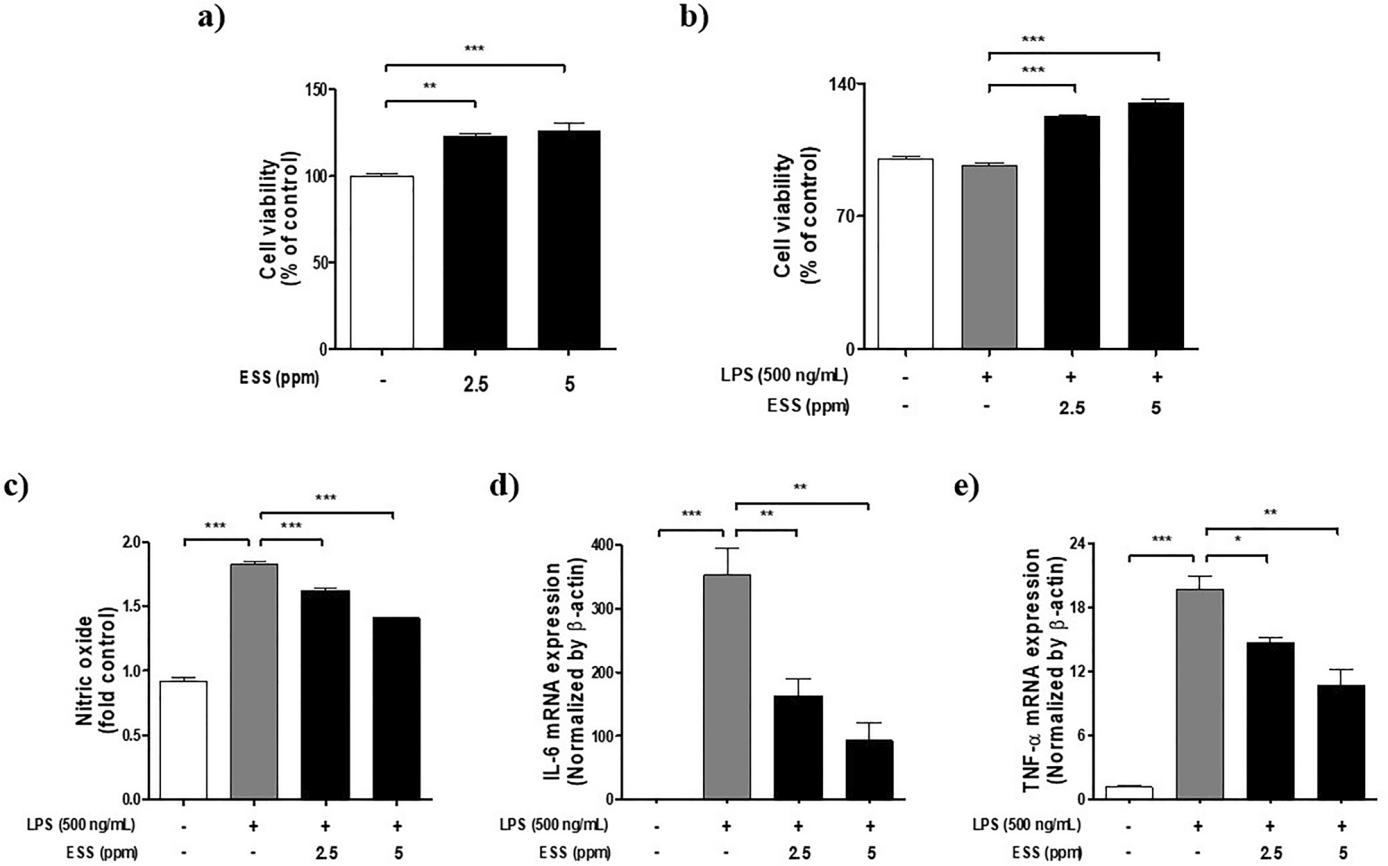
The effect of ESS on cell viability and anti-inflammatory responses in Raw 264.7 cells. The cell viability of RAW 264.7 cells was assessed by (**a**) treating with concentrations of ESS (2.5, 5 ppm) for 24 h and (**b**) pretreating with concentrations of ESS (2.5, 5 ppm) for 1 h, followed by treatment with LPS 500 ng/mL for 24 h. Based on the cell viability result, the anti-inflammatory effects of ESS on (**c**) No assay, (**d**) IL-6, and (**e**) TNF-α mRNA expression were determined. The RT-PCR analysis for RAW 264.7 cells was conducted in 12-well plates (4 × 10^5^ cells/well), with cells at 80% confluency pretreated with concentrations of ESS (2.5, 5 ppm) for 1 h, followed by treatment with LPS at 500 ng/mL for 6 h. The values presented are the mean ± SEM obtained from at least three independent experiments. Statistical significance is indicated as follows: ***p < 0.001, **p < 0.01, and *p < 0.05.

Based on RNA-seq, we investigated the anti-inflammatory effect of ESS on RAW 264.7 cell’s inflammatory response. An MTS assay was performed to determine the non-toxic concentration of ESS in Raw 264.7 cells with or without LPS (500 ng/mL), revealing an increase in cell viability up to 5 ppm in both conditions (Fig. 4a,b). Based on the results of cell viability, we conducted a NO assay. Upon LPS treatment, NO production increased by 90%, whereas with ESS treatment, there was a reduction of 20% at 2.5 ppm and 41% at 5 ppm. This indicates that ESS can effectively reduce the elevated levels of NO induced by LPS stimulation (Fig. 4c). Additionally, RT-PCR analysis showed that interleukin(IL)-6 and TNF-α mRNA expression levels, increased by LPS (500 ng/mL), were decreased in a concentration-dependent manner of ESS (Fig. 4d,e). These results suggest that ESS suppressed inflammation activity in LPS-induced macrophages in RAW 264.7 cells.

### Effects of ESS on cell viability and inflammatory cytokines in TNF-α-induced HaCaT cells

**Figure 5:**
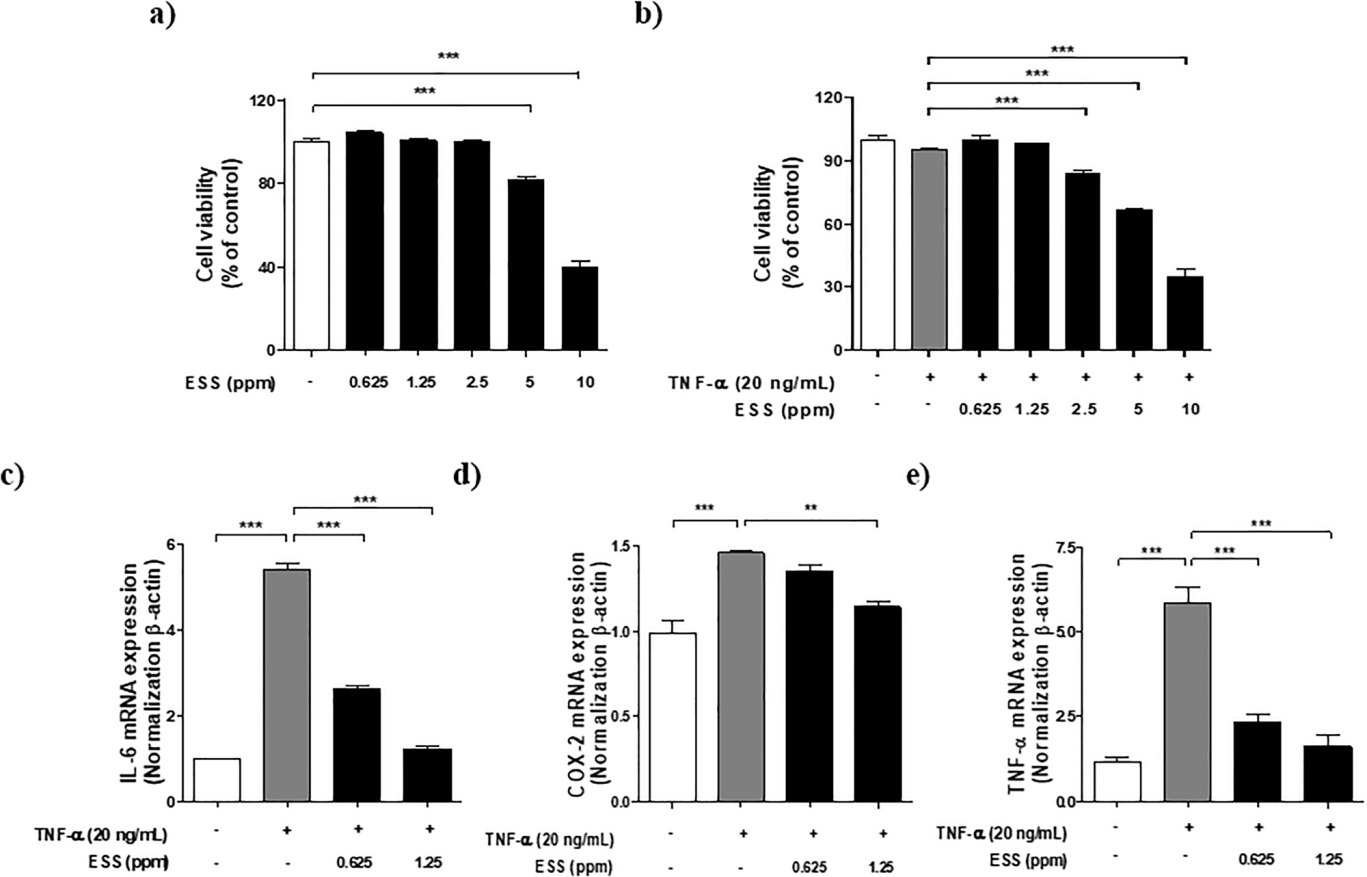
The effect of ESS on cell viability and anti-inflammatory effects in HaCaT cells. The cell viability of HaCaT cells was assessed by (**a**) treating with various concentrations of ESS (0.625, 1.25, 2.5, 5, 10 ppm) for 24 h and (**b**) pretreating with various concentrations of ESS (0.625, 1.25, 2.5, 5, 10 ppm) for 1 h, followed by treatment with HaCaT 20 ng/mL for 24 h. Based on the cell viability result, the anti-inflammatory effects of ESS on c) IL-6, (**d**) COX-2, and (**e**) TNF-α mRNA expression were determined. The RT-PCR analysis for HaCaT cells was conducted in 6-well plates (0.8 × 10^5^ cells/well), with cells at 80% confluency pretreated with ESS (0.625, 1.25 ppm) for 1 h, followed by treatment with TNF-α 20 ng/mL for 24 h. The values presented are the mean ± SEM obtained from at least three independent experiments. Statistical significance is indicated as follows: ***p < 0.001, **p < 0.01, and *p < 0.05.

Keratinocytes, one of the major cell types in the human epidermis, are involved in skin barrier functions. When activated by external stimuli, they release various inflammatory mediators and pro-inflammatory cytokines, leading to chronic inflammatory dermatitis such as atopic dermatitis^41^. In this study, we used the HaCaT keratinocyte cell line to investigate the effects of ESS on inflammation induced by TNF-α in HaCaT cells. To determine the non-toxic concentration of ESS in HaCaT cells, an MTS assay was performed, revealing no impact on cell viability up to 2.5 ppm (Fig. 5a). Next, an MTS assay was conducted on HaCaT cells with inflammation induced by TNF-α (20 ng/mL) to assess the effects of ESS, showing no impact on cell viability up to 1.25 ppm (Fig. 5b). Based on these findings, RT-PCR was performed to examine the influence of ESS on mRNA expression levels of inflammatory cytokines (IL-6, cyclooxygenase(COX)-2, TNF-α) in HaCaT cells with inflammation induced by TNF-α (20 ng/mL). After TNF-α stimulation, mRNA expression levels of inflammatory cytokines increased. However, in the ESS treatment, the mRNA expression levels of IL-6 and TNF-α decreased significantly in a concentration-dependent manner, and the level of COX-2 mRNA expression decreased at ESS 1.25 ppm (Fig. 5c–e). These results suggest that ESS could contribute to the suppression of inflammation response in keratinocytes induced by TNF-α in HaCaT cells.

### Effect of ESS on atopic dermatitis-like symptoms in BALB/c mice induced by DNCB

**Figure 6:**
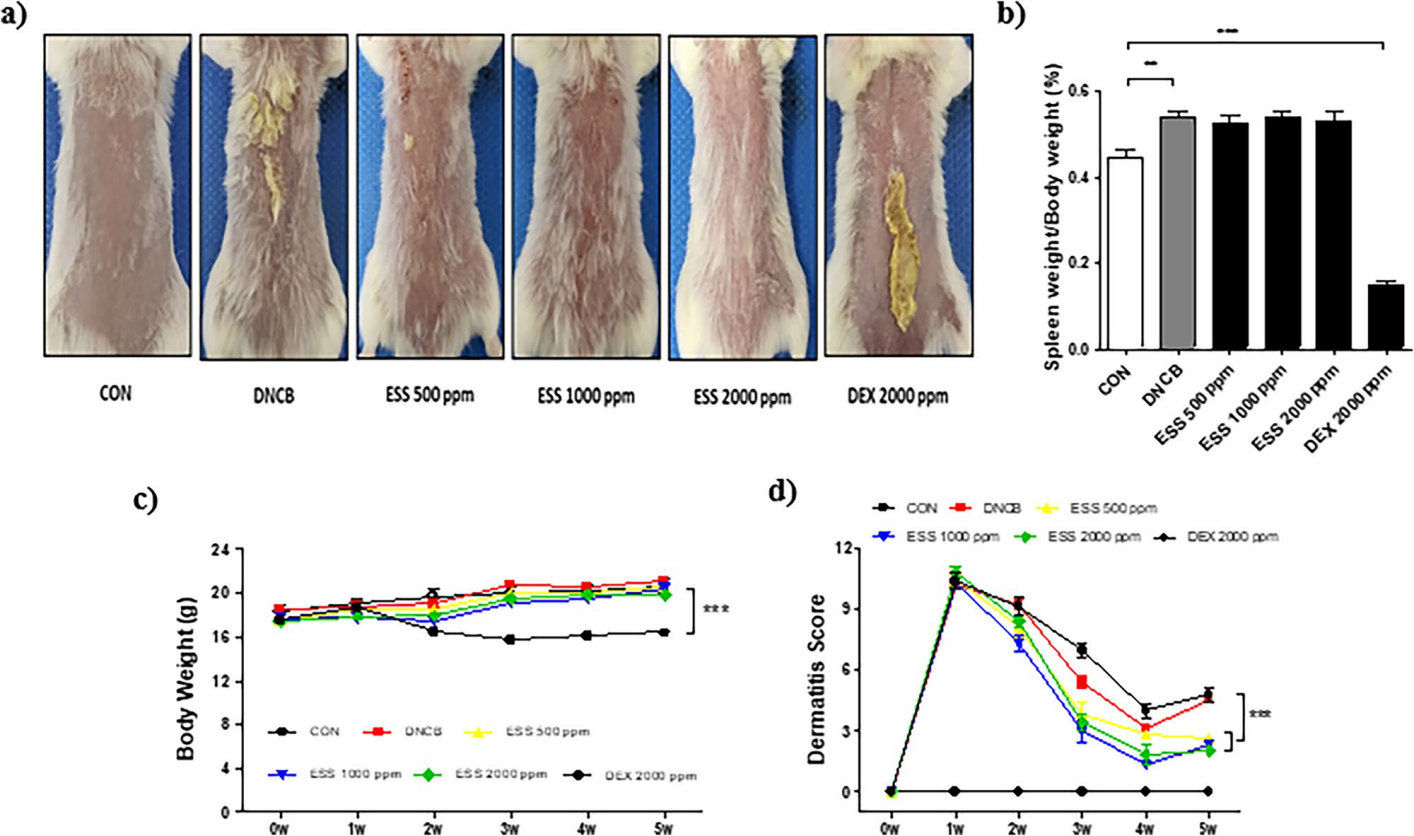
Improvement of skin conditions and dermatitis severity in DNCB-induced BALB/c mice by ESS. DNCB-induced BALB/c mice were topically treated with various concentrations of ESS (500, 1000, 2000 ppm) and DEX 2000 ppm (n = 5–8/group). On the final day of the experiment, (**a**) skin conditions of BALB/c mice were observed and (**b**) spleen weights were measured. During the experiment, (**c**) body weight and (**d**) dermatitis scores (based on four criteria: erythema/bleeding, edema, abrasion/erosion, and scale/dryness) were measured once a week. The values presented are the mean ± SEM obtained from at least five independent experiments. Statistical significance is indicated as follows: ***p < 0.001, **p < 0.01, and *p < 0.05.

Given that BALB/c mice exhibit symptoms similar to human atopic dermatitis when treated with DNCB, including clinical manifestations such as epidermal hyperplasia, pruritus, and scratching behavior^29,30^, we evaluated the effect of ESS in an atopic dermatitis mouse model based on existing data demonstrating its strong anti-inflammatory activity in macrophages and keratinocytes. After inducing atopic dermatitis-like symptoms with DNCB in BALB/c mice, we administered ESS topically to the mouse skin at different concentrations (500, 1000, 2000 ppm). Additionally, as a positive control, we topically applied Dexamethasone, a widely recognized anti-inflammatory and immunosuppressive agent. The skin conditions including the overproduction of stratum corneum were then visually observed. The skin condition improved with ESS treatment, particularly showing remarkable improvement at 2000 ppm. In contrast, treatment with dexamethasone showed limited efficacy (Fig. 6a). At the end of the experiment, spleen weight measurements revealed an increase in the DNCB group compared to the CON group, indicating splenomegaly, a hallmark of systemic immune diseases. However, there was no notable difference between the ESS group and the DNCB group, suggesting that ESS treatment had a limited impact on the splenomegaly induced by DNCB treatment (Fig. 6b). These results indicate that ESS treatment had a limited effect on spleen weight. Additionally, during the experimental period, the body weight of BALB/c mice and the severity of dermatitis were monitored once a week. There was no significant difference in body weight between the control and ESS groups compared to the DNCB group. However, the severity of dermatitis was significantly improved in the ESS group compared to the DNCB group (Fig. 6c,d). This suggests that ESS treatment led to an improvement in atopic dermatitis-like symptoms in the skin. On the other hand, the positive control group treated with DEX 2000 ppm showed a significant decrease in spleen weight compared to the CON group, and body weight loss was observed from the 2nd week of the experiment (Fig. 6b-c). Furthermore, the severity of dermatitis did not improve with dexamethasone treatment (Fig. 6d). In conclusion, our results demonstrate that topical application of ESS is effective in ameliorating DNCB-induced atopic dermatitis-like symptoms in BALB/c mice. In contrast, topical application of dexamethasone showed limited effectiveness in this model.

### Effect of ESS on histological changes of skin in DNCB-induced atopic-like dermatitis BALB/c mice

**Figure 7:**
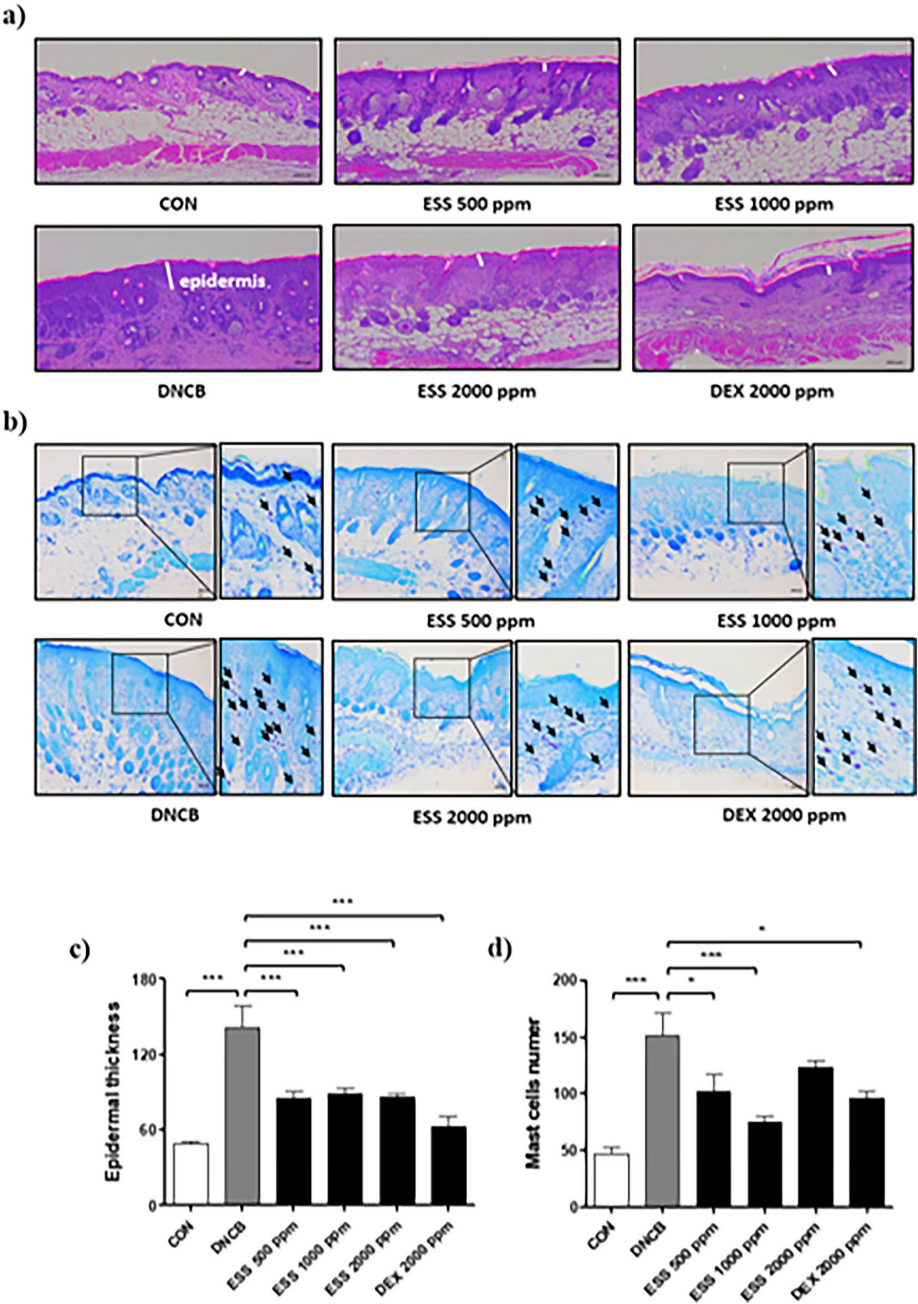
Skin histological improvement by ESS treatment in DNCB-induced BALB/c mice. In DNCB-induced BALB/c mice, we observed histopathological features of the skin that were altered by ESS treatment. Changes in epidermal thickness of the back skin induced by atopic dermatitis-like symptoms were observed with (**a**, **c**) H&E staining (n = 12 pictures per group), and changes in dermal infiltration of mast cells were observed with (**b**, **d**) Toluidine Blue staining (n = 20 pictures per group). The values presented are the mean ± SEM from independent experiments. Statistical significance is indicated as follows: ***p < 0.001, **p < 0.01, and *p < 0.05. Scale bar = 100 μm.

In atopic dermatitis, the impairment of the skin barrier and the excessive activation of immune responses lead to histological changes in skin tissue through inflammatory reactions within the skin^42^. To assess the impact of ESS on skin histopathological changes in DNCB-induced BALB/c mice, we conducted H&E staining and Toluidine blue staining using dorsal skin. The results of H&E staining revealed that the epidermal thickness of the DNCB group increased by 188% compared to the CON group. Compared to the DNCB group, the epidermal thickness decreased by 114%, 106%, and 112% in the ESS groups (500, 1000, and 2000 ppm), respectively, and the DEX group showed a decrease of 161% (Fig. 7a–c). Additionally, the observations from Toluidine blue staining indicated that the dermal infiltration of mast cells in the DNCB group increased by 221% compared to the CON group. Compared to the DNCB group, the dermal infiltration of mast cells in the ESS groups (500, 1000, and 2000 ppm) decreased by 104, 162, and 60%, respectively, and the DEX group exhibited a decrease of 117% (Fig. 7b–d). These findings indicate that the local application of ESS improves the histopathological changes of atopic dermatitis, suggesting its potential in alleviating skin inflammation.

### Effect of ESS on atopic dermatitis-like symptoms in HR-1 mice induced by DNCB

**Figure 8:**
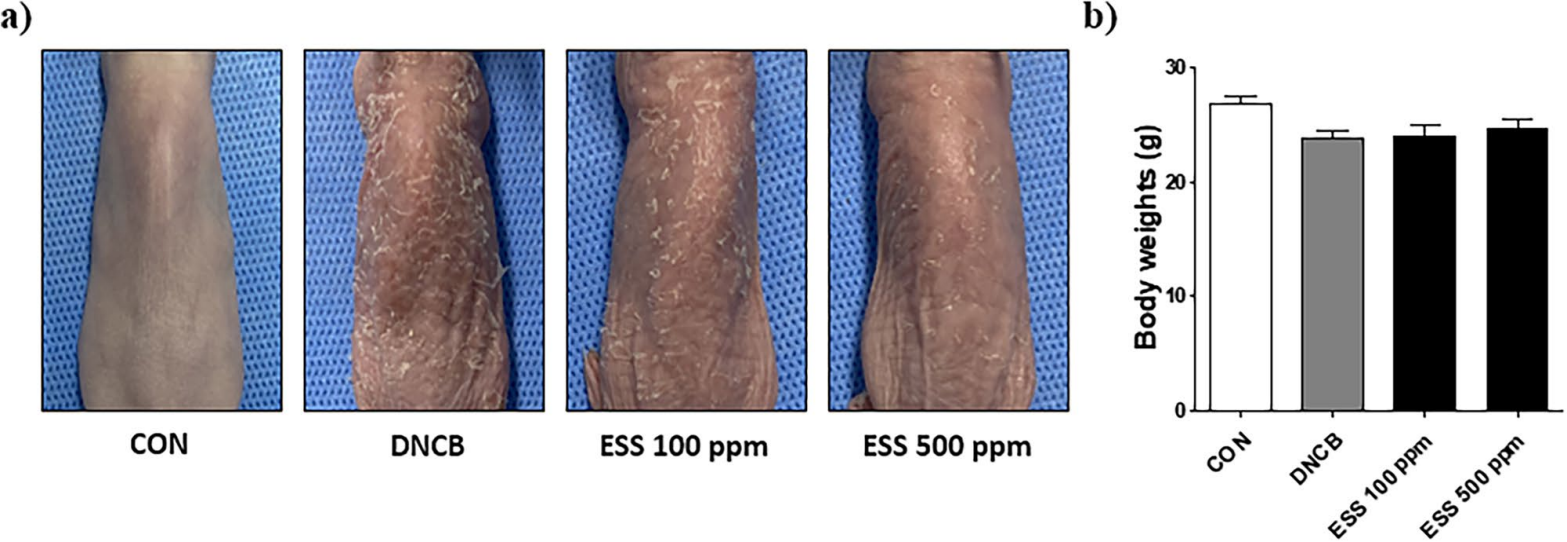
Improvement effect of skin conditions in DNCB-induced HR-1 mice by ESS. DNCB-induced HR-1 mice were topically treated with various concentrations of ESS (100, 500 ppm) (n = 7/group). On the final day of the experiment, (**a**) the skin conditions of HR-1 mice were observed and (**b**) body weight was measured. The values presented are the mean ± SEM obtained from at least seven independent experiments. Statistical significance is indicated as follows: ***p < 0.001, **p < 0.01, and *p < 0.05.

Based on the previous experimental data showing the beneficial effects of ESS on atopic dermatitis-like symptoms in DNCB-induced BALB/c mice, we conducted an additional experiment using HR-1 hairless mice as a substitute, which are widely used in transdermal drug penetration studies as a human skin model^28^. Unlike BALB/c mice, HR-1 hairless mice did not develop atopic dermatitis-like symptoms with short-term DNCB treatment, so we induced atopic dermatitis-like symptoms through long-term DNCB treatment for the experiment. Considering the ineffective results of topical dexamethasone application in previous trials, we excluded the dexamethasone group from this study. After topically applying ESS to the dorsal skin of DNCB-induced HR-1 hairless mice, we visually observed the skin condition, including excessive stratum corneum formation and scratch wounds due to pruritus. The results showed that various concentrations of ESS (100, 500 ppm) improved the skin condition, with particularly noticeable improvement observed at 500 ppm (Fig. 8a). At the end of the experiment, there were no significant changes in body weight observed in the DNCB and ESS groups compared to the CON group in HR-1 hairless mice (Fig. 8b). This suggests that ESS treatment had little effect on the body weight of the mice.

### Effects of ESS on histological changes and serum IgE levels in HR-1 mice with DNCB-induced atopic dermatitis-like symptoms

**Figure 9:**
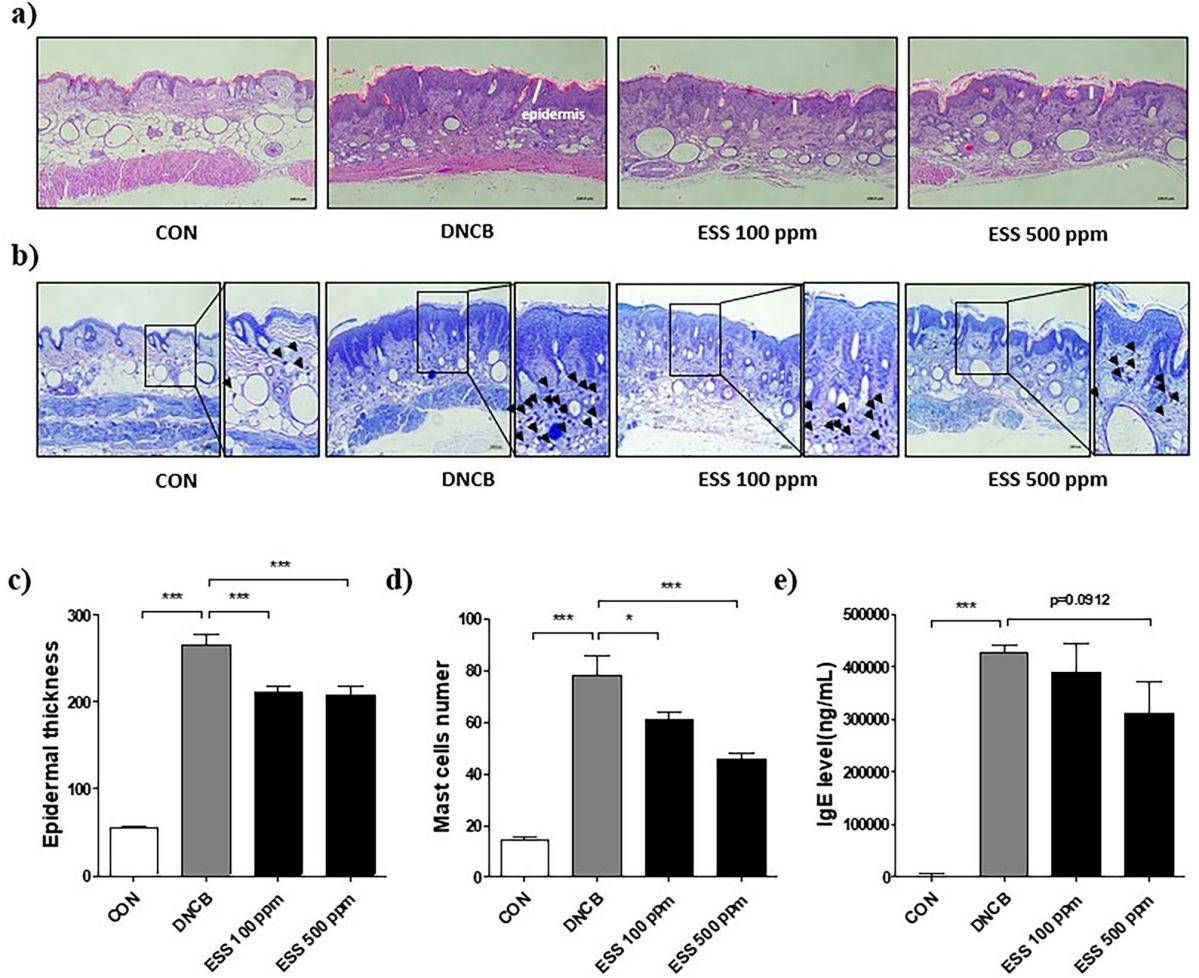
Improvement of skin histology and serum IgE by ESS treatment in DNCB-induced HR-1 mice. In DNCB-induced HR-1 mice, we observed histopathological features of the skin that were altered by ESS treatment. Changes in epidermal thickness of the back skin induced by atopic dermatitis-like symptoms were observed with (**a**, **c**) H&E staining (n = 12 pictures per group), and changes in dermal infiltration of mast cells were observed with (**b**, **d**) Toluidine Blue staining (n = 20 pictures per group). (**e**) The effect of ESS treatment on serum IgE levels in HR-1 mice with atopic dermatitis-like symptoms DNCB-induced (n = 7 per group). The values presented are the mean ± SEM from independent experiments. Statistical significance is indicated as follows: ***p < 0.001, **p < 0.01, and *p < 0.05. Scale bar = 100 μm.

To assess the impact of ESS on skin histological changes in DNCB-induced HR-1 mice, we conducted H&E staining and Toluidine blue staining of the dorsal skin. The results from H&E staining revealed significant changes in the epidermal thickness of the dorsal skin. In comparison to the CON group, the DNCB group exhibited a remarkable 376% increase in epidermal thickness. Compared to the DNCB group, the epidermal thickness decreased by 98 and 104% in the ESS groups (100, and 500 ppm), respectively (Fig. 9a–c). Additionally, Toluidine blue staining demonstrated shifts in mast cell infiltration within the dermal layer of the dorsal skin. Specifically, the DNCB group displayed a substantial 431% increase in mast cell infiltration compared to the CON group. Compared to the DNCB group, the mast cell infiltration decreased by 115 and 221% in the ESS groups (100, and 500 ppm), respectively (Fig. 9b–d). Furthermore, the measurement of serum IgE levels, a key marker of atopic dermatitis, indicated a tendency of decreased IgE levels in the ESS 500 ppm group, compared to the increase observed in the DNCB group (Fig. 9e). These findings even indicate that ESS, in HR-1 mice as well, has the potential to ameliorate the histopathological changes of atopic dermatitis and improve the state of skin inflammation.

### Effects of ESS on serum cytokines in HR-1 mice with DNCB-induced atopic dermatitis-like symptoms

**Figure 10:**
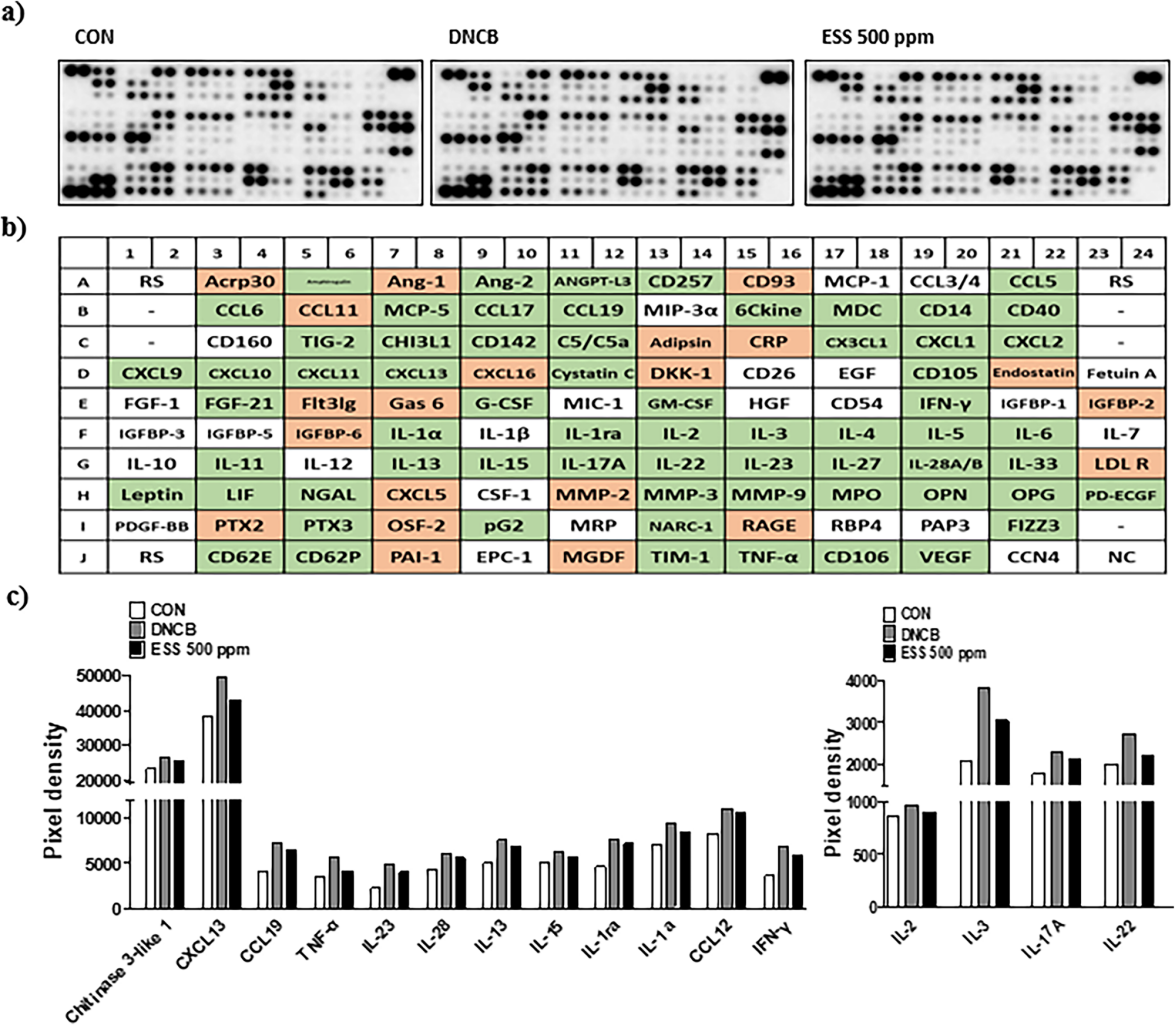
Improvement of inflammation-related cytokines in HR-1 mouse serum by ESS treatment. To investigate the effect of ESS treatment on cytokines in DNCB-induced HR-1 mice, (**a**) 111 cytokines were measured using the Proteome Profiler Mouse XL cytokine array. In the figure, (**b**) green indicates cytokines that decreased in the ESS group compared to the DNCB group, while orange represents cytokines that increased in the ESS group compared to the DNCB group. (**c**) Among the 111 altered cytokines, changes in inflammation-related cytokines are highlighted.

Based on the observed trend of decreased IgE levels in the ESS 500 ppm group, we investigated the impact of ESS 500 ppm on serum cytokines in DNCB-induced HR-1 mice. Using the Proteome Profiler Mouse XL cytokine array, we screened a total of 111 cytokines and identified changes in 85 cytokines (Fig. 10a,b). Among these, inflammation-associated cytokines (Chitinase 3-like(Chi3l) 1, CXCL13, CCL19, TNF-α, IL-23, IL-28, IL-13, IL-15, IL-1ra, IL-1α, CCL12, Complement component C5a, Interferon gamma(IFN)-γ, IL-2, IL-3, IL-17a, IL-22) that increased due to DNCB treatment showed a tendency to decrease in the ESS 500 ppm treated group (Fig. 10c). These results indicate that ESS may regulate inflammatory signals in atopic dermatitis-like symptoms, contributing to the improvement of skin conditions.

### Effects of ESS on mRNA levels of inflammation-related genes based on microarray of DNCB-induced HR-1 mouse skin

In previous studies utilizing DNCB-induced atopic dermatitis-like symptoms mouse models, inflammatory cytokines such as TNF-α, COX-2, IL-6, IL-13, IL-31, IL-15, IFN-γ, thymus and activation-regulated chemokine(TARC), and thymic stromal lymphopoietin (TSLP), and others have been widely recognized as markers of atopic dermatitis in the skin^1,26,42^. However, our research showed that DNCB treatment did not upregulate these inflammatory cytokine genes under our experimental conditions possibly due to the prolonged treatment of DNCB (Figure S2). Nevertheless, it is important to note that other markers associated with atopic dermatitis, such as epidermal thickness and mast cell infiltration, notably increased in response to DNCB treatment. These findings indicate that in our specific model, the traditional inflammatory cytokine markers may not be significantly affected, while other relevant indicators of atopic dermatitis remain evident. It is important to highlight that classical inflammatory markers such as TNF-α, IL-6, etc., may not consistently serve as indicators for atopic dermatitis induced by DNCB. The choice of marker genes can vary based on experimental conditions, including the duration of DNCB treatment, the manifestation of skin symptoms, mouse models utilized, the severity of atopic dermatitis, and other pertinent factors. Further investigation is necessary to fully elucidate the underlying mechanisms and implications of these intriguing observations.

**Figure 11:**
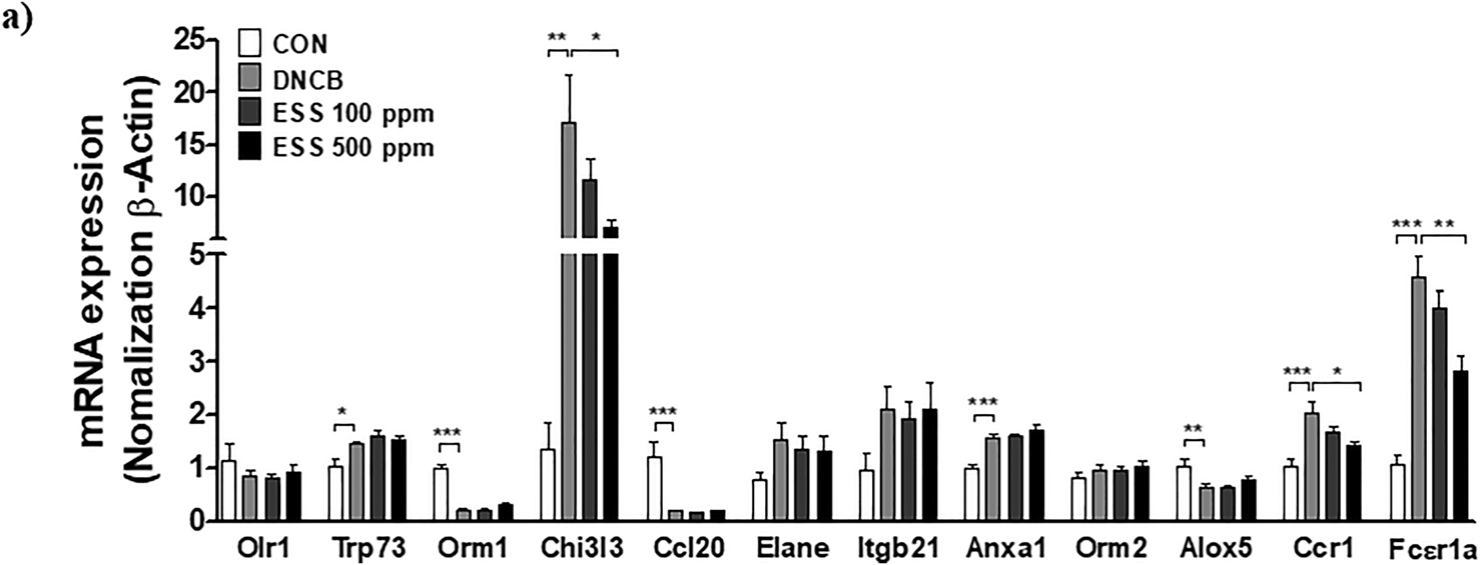
Improvement of inflammation-related gene expression in HR-1 mouse skin by ESS treatment. (**a**) The modulation of inflammation-related gene expression in DNCB-induced HR-1 mouse skin by ESS treatment was investigated through RT-PCR. The values presented are the mean ± SEM obtained from at least seven independent experiments. Statistical significance is indicated as follows: ***p < 0.001, **p < 0.01, and *p < 0.05.

According to recent research, cDNA microarray analysis of DNCB-induced mouse skin revealed an increase in 30 inflammation-related genes in the atopic dermatitis-like symptoms group compared to the non-induced group^39^. Based on this study, we selected the top 12 genes among them and confirmed their mRNA expression changes using RT-PCR. As a result, the mRNA expression levels of transformation related protein(Trp)73, Chi3l3, annexin A1(Anxa1), C–C chemokine receptor(Ccr)1, and Fc receptor, IgE, high affinity I, alpha peptide (Fcεr1a) were significantly increased by DNCB, while orosomucoid(Orm)-1, Ccl20, and 5-lipoxygenase(Alox5) mRNA expression levels were significantly decreased by DNCB. Among them, the mRNA expression levels of Chi3l3, Ccr1, and Fcεr1a, which were increased by DNCB, were found to decrease in the ESS 500 ppm group (Fig. 11a). Chi3l3 and Ccr1 play important roles in regulating skin inflammation, and the Fcεr1a gene is crucial in controlling immune responses mediated by IgE^43–45^. Additionally, Anxa1 regulates T cell activity in skin inflammation, and Ccl20 and Trp73 are involved in inflammatory responses^46–48^. Orm-1 acts as an anti-inflammatory protein regulating immune responses, and Alox5 induces cell death ^49,50^. The changes in these genes are associated with the complex occurrence and regulation mechanisms of skin inflammation, particularly playing crucial roles in the inflammatory process. However, the roles of most genes in atopic dermatitis remain unclear, indicating the need for further research. Nonetheless, These findings demonstrate that those genes may be more reliable markers of DNCB-induced atopic dermatitis-like symptoms in our experimental conditions and ESS also improves the increased mRNA expression levels of these genes induced by DNCB. Together, the data implies the potential of ESS to modulate the inflammatory response and contribute to the improvement of skin conditions in atopic dermatitis-like symptoms.

### Effects of ESS main compounds, SCM, SQA, and SHQA, on the inflammatory response in LPS-induced Raw 264.7 cells

**Figure 12:**
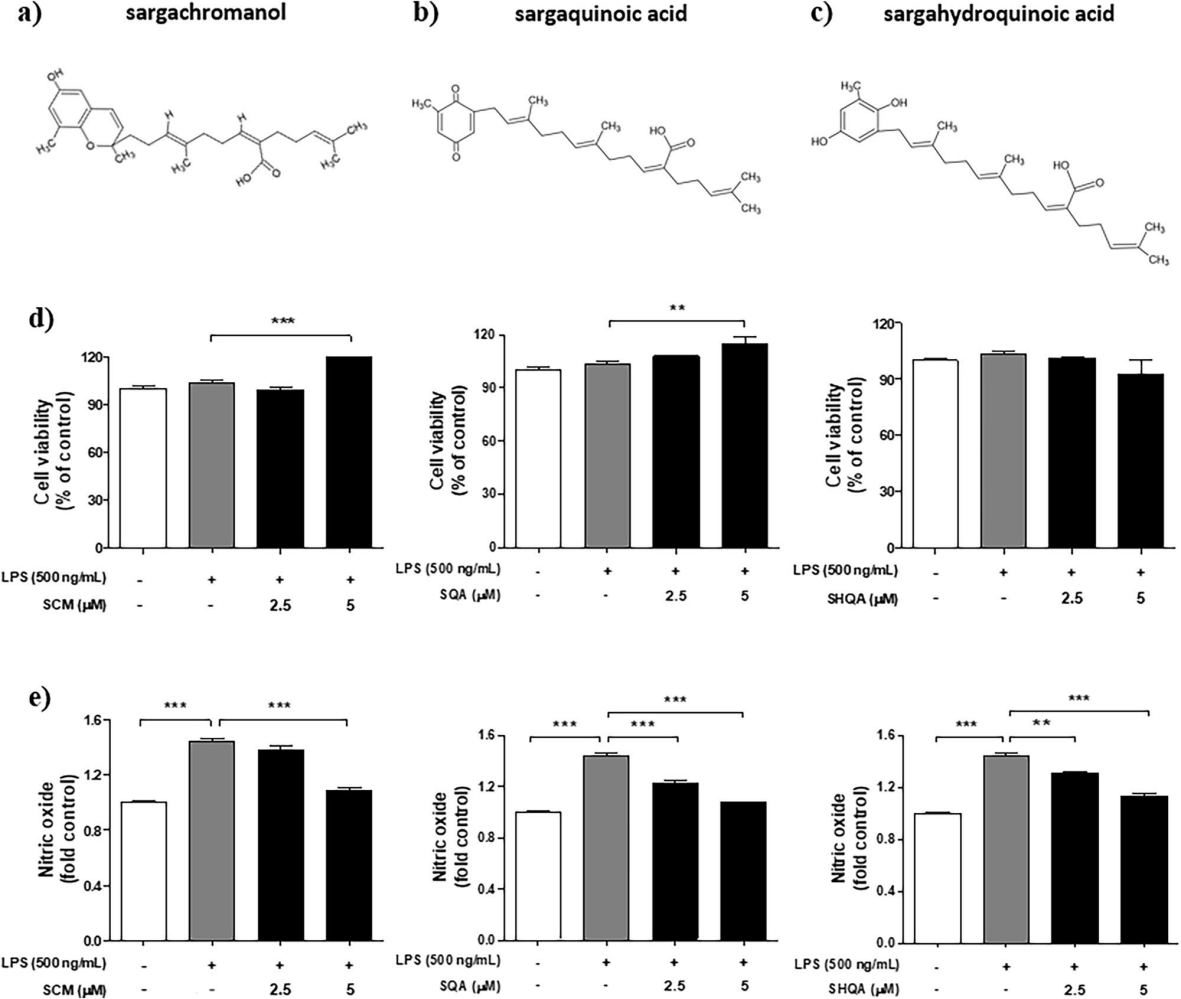
The structure of ESS compounds (SCM, SQA, SHQA) and anti-inflammatory effects in Raw 264.7 cells. The structure of (**a**) sargachromanol (SCM, PubChem CID : 10455044), (**b**) sargaquinoic acid (SQA, PubChem CID : 101145056), and (**c**) sargahydroquinoic acid (SHQA, PubChem CID : 10202734), the key components of ESS. The cell viability of RAW 264.7 cells was assessed by pretreating with concentrations of (**d**) SCM (2.5, 5 μM), SQA (2.5, 5 μM), SHQA (2.5, 5 μM) for 1 h, followed by treatment with LPS 500 ng/mL for 24 h. Based on the cell viability result, the anti-inflammatory effects of e) SCM, SQA, and SHQA were measured using the NO assay. The values presented are the mean ± SEM obtained from at least three independent experiments. Statistical significance is indicated as follows: ***p < 0.001, **p < 0.01.

Based on the previous cell and animal experiments, we confirmed the anti-inflammatory effects and the improvement in atopic dermatitis-like symptoms by ESS. Many studies indicate that ESS is primarily composed of key compounds well-known for their biological activities, such as sargachromanol (SCM), sargaquinoic acid (SQA), and sargahydroquinoic acid (SHQA) (Fig. 12a–c)^50,51^. To determine which compound contributes more significantly to the improvement of atopic dermatitis-like symptoms, we evaluated the anti-inflammatory effects of each compound using the RAW 264.7 macrophage cell line. First, we conducted an MTS assay to evaluate how SCM, SQA, and SHQA affected Raw 264.7 cells induced with inflammation via LPS (500 ng/mL) stimulation. The results showed that SCM and SQA increased cell viability at a concentration of 5 ppm, while SHQA showed no toxicity up to 5 ppm (Fig. 12d). Based on these results, we conducted a NO assay to determine the impact of these compounds on the elevated NO production induced by LPS (500 ng/mL). The results revealed that SCM, at a concentration of 5 ppm, and SQA and SHQA, in a concentration-dependent manner, significantly reduced NO production (Fig. 12e). These findings suggest that SCM, SQA, and SHQA may synergistically contribute to the suppression of inflammatory activity in LPS-induced macrophages.

## Discussion

Atopic dermatitis is a complex inflammatory skin disorder with a significant worldwide prevalence, presenting a challenge for effective management and patient well-being. This study explores the potential benefits of utilizing ethanolic extract of *Sargassum serratifolium* (ESS) as an approach to treating atopic dermatitis, examining both in vitro cell line models and in vivo mouse models^52^. Based on the anti-inflammatory effects observed in ESS’s in vitro cell line model, we observed improvements in both skin morphological conditions including overproduction of stratum corneum and skin inflammation responses in the mouse model with induced atopic dermatitis-like symptoms. These observations contribute to an increased understanding of not only atopic dermatitis but also the potential applications of marine-derived compounds in skincare management.

The ethanolic extract of *Sargassum serratifolium* used in our study comes from *Sargassum serratifolium* which is not commonly used, even though it’s edible in Asian countries. UN has identified *Sargassum* species as pollutants in marine ecosystems due to their excessive growth^12–14^. Our research shows that this seaweed extract holds promise as a therapeutic agent for managing atopic dermatitis, offering a unique solution to repurpose marine resources for medical purposes.

In terms of economics, using ESS as a therapeutic agent for atopic dermatitis could potentially reduce healthcare costs associated with disease management. Conventional treatments often come with high costs and potential side effects, placing financial strain on patients and healthcare systems. Mild to moderate atopic dermatitis is typically managed using local treatments. Initial management commonly involves the use of corticosteroids and immunosuppressants. Short-term and intermittent use of corticosteroids effectively alleviates symptoms and enhances the patient’s quality of life. However, when corticosteroids are used for an extended period, they improve inflammatory responses in the skin, while simultaneously inhibiting the proliferation and differentiation of keratinocytes, ultimately leading to adverse effects such as skin atrophy, severe inflammation, erythema, edema, and the formation of scales^53,54^. In our experimental conditions, the decrease in epidermal thickness and reduction in infiltrating mast cells in the dermis observed upon dexamethasone treatment are considered as phenomena that can occur due to prolonged local corticosteroid application. Additionally, considering the visual skin conditions such as weight loss and excessive scale formation, dexamethasone under our experimental conditions did not ameliorate atopic dermatitis-like symptoms induced by DNCB. Conversely, ESS demonstrated its efficacy in improving atopic dermatitis-like symptoms in the same experiments including dermatitis score, body weight, visual observation, etc. These findings suggest that ESS could serve as an alternative treatment option for atopic dermatitis, opening up new possibilities in therapeutic approaches. By introducing a natural, cost-effective alternative derived from marine resources, we pave the way for more sustainable and affordable treatment options, potentially reducing healthcare expenses and improving access to care.

The RNA sequencing analysis of Raw 264.7 macrophages stimulated by LPS reveals the multi-faceted regulatory potential of ESS on inflammatory responses. Through comprehensive differential gene expression analysis, we identified 2022 genes altered in the LPS vs. LPS + ESS treatment groups, encompassing both upregulation and downregulation. To further elucidate the underlying regulatory mechanisms, we conducted a KEGG enrichment analysis on the differentially expressed genes in the LPS + ESS group. This analysis highlighted the involvement of various signaling pathways associated with inflammatory responses, including TNF, NF-kappa B, and Toll-like receptor pathways. Subsequently, we performed protein–protein interaction analysis using the STRING database. Our findings revealed highly interconnected protein–protein interactions within the TNF, NF-kappa B, and Toll-like receptor signaling pathways. This complex network suggests that ESS may exert its regulatory effects by modulating the expression of various cytokines and chemokines involved in inflammatory responses in macrophages. However, further studies are required to unravel the detailed mechanisms underlying ESS-mediated anti-atopic dermatitis effects, considering the involvement of diverse immune and skin cells in the symptoms of this disease.

Based on these findings, we confirmed the anti-inflammatory effects of ESS in LPS-treated Raw 264.7 macrophage and TNF-α treated HaCaT keratinocytes as models of inflammation-induced cells. Our results showed that ESS inhibits NO production associated with macrophage activation and atopic dermatitis pathology and significantly reduces mRNA expression of inflammation-related cytokines in both cell models. The consistent regulation of inflammatory responses observed in both cell models emphasizes the promising potential of ESS as a multifaceted therapeutic for inflammatory skin conditions.

It should be noted that although we demonstrated the anti-inflammatory properties of the ethanolic extract of ESS using RAW 264.7 cells, these cells differ significantly from actual macrophages in the skin. Starting early in life, organs harbor monocyte-derived macrophages (MDMs) that can either become long-lived or be replenished from bone marrow hematopoietic stem cells^55^. Despite prenatal seeding of dermal macrophages from fetal sources, studies show that dermal macrophages are partially replaced by MDMs postnatally^56,57^.

A study highlighted differences between primary macrophages and RAW 264.7 cells in TLR4 and NF-κB activation kinetics. NF-κB activation in primary macrophages occurs much faster than in RAW 264.7 cells. Post-LPS activation, NF-κB phosphorylation in primary macrophages peaked at 5 min and nuclear translocation at 10 min, while in RAW 264.7 cells, these processes peaked at 30 min. Low LPS concentrations induced slower responses, but primary macrophages responded faster (50 min) compared to RAW cells (75 min)^58^. Mathematical modeling showed that higher baseline NF-κB levels in primary macrophages speed up NF-κB translocation. Baseline nuclear NF-κB levels were 25–35% in primary macrophages, compared to 5–10% in RAW 264.7 cells. This rapid response mechanism may be crucial for pathogen detection at low LPS concentrations^58^. Based on this study, RAW 264.7 macrophages may not fully mimic skin-resident macrophages, suggesting that further studies should be performed using primary cultured macrophages to elucidate the beneficial roles of ESS on macrophages. Skin-resident macrophages, derived from both fetal sources and postnatal bone marrow, may exhibit distinct physiological and functional characteristics that are not completely replicated by the RAW 264.7 cell line. Primary cultured macrophages derived directly from skin tissue would provide a more accurate representation of the in vivo environment, enabling a better understanding of how ESS influences macrophage behavior and inflammatory processes in the skin.

Furthermore, primary macrophages possess a unique gene expression profile and signaling pathway activation kinetics that differ from transformed cell lines^58^. These differences are crucial when investigating the molecular mechanisms underlying the anti-inflammatory effects of ESS. For instance, the faster NF-κB activation and nuclear translocation observed in primary macrophages compared to RAW 264.7 cells^58^ suggest that ESS may modulate these pathways differently in a primary cell context. Evaluating the impact of ESS on primary skin macrophages would likely yield insights into its precise mechanisms of action, including its effects on cytokine production, phagocytosis, and interaction with other immune cells. This approach would provide a more comprehensive understanding of how ESS may be beneficial in treating inflammatory conditions in the skin.

The transition to in vivo models not only enhances the study’s practical implications but also bolsters the credibility of its findings. The application of ESS on mouse models displaying atopic dermatitis-like symptoms induced by DNCB resulted in the improvement of skin condition, confirmed through visual observation. In the case of BALB/c mice, ESS convincingly demonstrates its potential for alleviating the distressing symptoms of atopic dermatitis, substantiated not only through visual observation but also by a decrease in dermatitis score (erythema/hemorrhage, edema, excoriation/erosion and scaling/dryness). Moreover, observations regarding changes in spleen weight due to ESS treatment suggest that ESS had a limited impact on splenomegaly induced by DNCB. This indicates that the influence of ESS may be confined to the skin, warranting further research, but also suggesting the potential for minimizing systemic effects.

A closer examination of the study’s findings through histological examination of skin tissue reveals subtle yet significant practical impacts of ESS treatment. The observed reduction in epidermal thickness in both animal models indicates that ESS reduced the excessive proliferation of keratinocytes induced by DNCB-triggered inflammatory responses^27^. Additionally, mast cells are activated cells through high-affinity IgE receptors, which, when activated, release granules, triggering allergic inflammatory responses^59,60^. Therefore, the decrease in dermal infiltration of mast cells signifies that ESS reduced allergic inflammatory responses induced by DNCB. The trend of improvement in IgE levels observed in the serum of HR-1 mice treated with ESS at 500 ppm supports the amelioration of symptoms resembling atopic dermatitis by reducing dermal mast cell infiltration. Furthermore, changes in serum inflammation-related cytokines observed in the DNCB-induced HR-1 mouse indicate the potential of ESS treatment in regulating various inflammatory signaling pathways in DNCB-induced atopic dermatitis-like symptoms. These histological changes, as well as the changes in serum IgE and cytokine levels, align with hypothesized alterations in inflammation and immune response, highlighting ESS’s potential involvement in the underlying cellular and molecular pathways associated with atopic dermatitis.

Building upon the findings of previous experiments, we observed the impact of ESS on atopic dermatitis-like symptoms from a molecular biology experiment. Under our experimental conditions, classical inflammatory markers (TNF-α, COX-2, IL-6, IL-13, IL-31, IL-15, IFN-γ, TARC, and TSLP) of atopic dermatitis did not show significant changes. However, there was a notable increase in other indicators of atopic dermatitis, such as skin condition improvement and histological changes, in response to DNCB treatment. This suggests the possibility of additional genetic markers beyond the known ones. Therefore, our study shifts focus to explore the less-investigated potential therapeutic efficacy of ESS in atopic dermatitis. In recent research, other researchers investigated specific inflammation-related genes associated with atopic dermatitis by conducting cDNA microarray analysis on mouse skin induced with atopic dermatitis-like symptoms using DNCB^39^. The results of the cDNA microarray analysis showed that 30 inflammation-related genes were upregulated by more than 2-fold in the DNCB group compared to the untreated group. This discovery is particularly noteworthy because the majority of these genes were not well-known in the context of previous research on atopic dermatitis^39^. Accordingly, through this research, we selected the top 12 genes obtained from cDNA microarray analysis and measured their mRNA expression levels using RT-PCR in the skin of DNCB-induced HR-1 mice. The results showed significant changes in the genes Trp73, Orm-1, Chi3l3, Ccl20, Anxa1, Alox5, Ccr1, and Fcεr1a upon DNCB treatment. Considering their pivotal roles in the complex initiation and regulation mechanisms of skin inflammation, or processes such as cell apoptosis and T cell activity regulation, we have become interested in exploring new pathways^43–50^. Additionally, contrasting the substantial decrease observed in the ESS 500 ppm treatment for Chi3l3, Ccr1, and Fcεr1a genes, they exhibited significant increases in the DNCB group. These outcomes underscore the potential of these genes as reliable markers for DNCB-induced atopic dermatitis-like symptoms under our experimental conditions and emphasize ESS’s modulatory capabilities. However, further additional research is needed to elucidate how these intriguing observations contribute to understanding the underlying mechanisms and significance related to atopic dermatitis. Through these observations, we anticipate gaining deeper insights into the complex interactions among the mechanisms of atopic dermatitis and associated biomolecules.

Langerhans cells (LC) indeed play a central role in atopic dermatitis, particularly in immune dysregulation and inflammatory responses, serving as key players in the pathogenesis of the condition. LC are recruited and activated by various pro-inflammatory cytokines released from keratinocytes. Specifically, TSLP contributes significantly to the recruitment and activation of LC, promoting the secretion of chemokines and cytokines that attract Th2 cells^61,62^. However, it is crucial to emphasize that no significant changes were observed in classic factors of atopic dermatitis, such as TSLP and pro-inflammatory cytokines (TNF-α, COX-2, IL-6, IL-31, etc.), in our study. Therefore, rather than interpreting the results solely focusing on LC and classic factors of atopic dermatitis, our aim was to explore genetic factors that could serve as novel indicators of the disease, shedding light on less explored aspects and aiming for a deeper understanding of its underlying mechanisms. However, acknowledging the ongoing importance of LC in atopic dermatitis, additional research is needed to elucidate how significant genetic factors (Trp73, Orm-1, Chi3l3, Ccl20, AnXa1, Alox5, Ccr1, and Fcεr1a, etc.) identified in our study may interact with LC function and the disease process. These efforts will provide a more comprehensive insight into the complex interactions among these LC, thus enhancing our understanding of the pathophysiology of atopic dermatitis.

In this study, we confirmed the anti-inflammatory effects and improvement in atopic dermatitis-like symptoms by ESS. Therefore, we evaluated the roles of the key compounds of ESS (SCM, SQA, and SHQA) in anti-inflammatory effects. The results demonstrated anti-inflammatory effects for all components, particularly significant for SQA and SHQA, even at a low concentration of 2.5 μM. This underlines the substantial roles of SCM, SQA, and SHQA in curtailing inflammatory activity in RAW 264.7 cells, showcasing their contributions to ESS’s overall anti-inflammatory and improvement effects on atopic dermatitis-like symptoms. However, the specific contributions of each compound to atopic dermatitis-like symptoms improvement and how interactions among these three compounds affect this remain unclear. Hence, investigations at the chemical and biological levels are imperative to grasp the intricate mechanisms at play. Additionally, the outcomes obtained in this experimental setup necessitate further studies to confirm if similar responses manifest within the actual physiological environment. This emphasizes the need for continued research, significantly augmenting our comprehension of *Sargassum serratifolium* utilization and its role in treating atopic dermatitis from a broader perspective.

### Supplementary Information

Supplementary Information.

## Data Availability

All data generated or analysed during this study are included in this published article (and its Supplementary Information files).
