# Supplementary material for: The beneficial effects of ethanolic extract of Sargassum serratifolium in DNCB-induced mouse model of atopic dermatitis
Source: Sci Rep. 2024 Jun 5;14:12874. doi: 10.1038/s41598-024-62828-z (PMC11150456; doi:10.1038/s41598-024-62828-z)
Supplement: Supplementary file 1 — Supplementary Information. [file 41598_2024_62828_MOESM1_ESM.docx]

**Supplementary Information**

**Table S1. The mouse primer sequences used in RT-PCR**

| **Gene** | **Primer sequences** | |
| --- | --- | --- |
| IL-6 | **Forward** | CCC ACC AAG AAC GAT AGT CA |
|  | **Reverse** | CTC CGA CTT GTG AAG TGG TA |
| TNF-α | **Forward** | TGG AAC TGG CAG AAG AGG |
|  | **Reverse** | AGA CAG AAG AGC GTG GTG |
| COX-2 | **Forward** | ATC TGG CTT CGG GAG CAC AA |
|  | **Reverse** | GTG GTA ACC GCT CAG GTG TT |
| IL-13 | **Forward** | CCT GGC TCT TGC CCT T |
|  | **Reverse** | GGT CTT GTG TGA TGT TGC TCA |
| IL-31 | **Forward** | CCT ACC CTG GTG CGT CTT TG |
|  | **Reverse** | CTG ACA TCC CAG ATG CCT GC |
| IL-15 | **Forward** | TGA GGA ACG TGC TGT ACC TG |
|  | **Reverse** | GGT CTT CTC CTC CAG CTC CT |
| IFN-γ | **Forward** | TCA AGT GGC ATA GAT GTG GAA GAA |
|  | **Reverse** | TGG CTC TGC AGG ATT TTC ATG |
| TARC | **Forward** | TAC TAT ACT CTC AAT CCT ATC CCT |
|  | **Reverse** | ACT TCT TGT GCC ATT TCC TG |
| TSLP | **Forward** | AGA GAA GCC CTC AAT GAC CAT |
|  | **Reverse** | GGA CTT CTG TGC CAT TCC |

**Table S2. The human primer sequences used in RT-PCR**

| **Gene** | **Primer sequences** | |
| --- | --- | --- |
| IL-6 | **Forward** | AGG GCT CTT CGG GAA ATG T |
|  | **Reverse** | GAA GAA GGA ATG CCC ATT AAC AAC |
| COX-2 | **Forward** | AAG CAG GCT AAT ACT GAT AGG |
|  | **Reverse** | TGT TGA AAA GTA GTT CTG GG |
| TNF-α | **Forward** | CTA TCT GGG AGG GGT CTT CC |
|  | **Reverse** | ATG TTC GTC CTC CTC ACA GG |

**Figure S1. Effect of ESS on TNF-α-induced phosphorylation of p38 and NF-κB in HaCaT cells.**

**
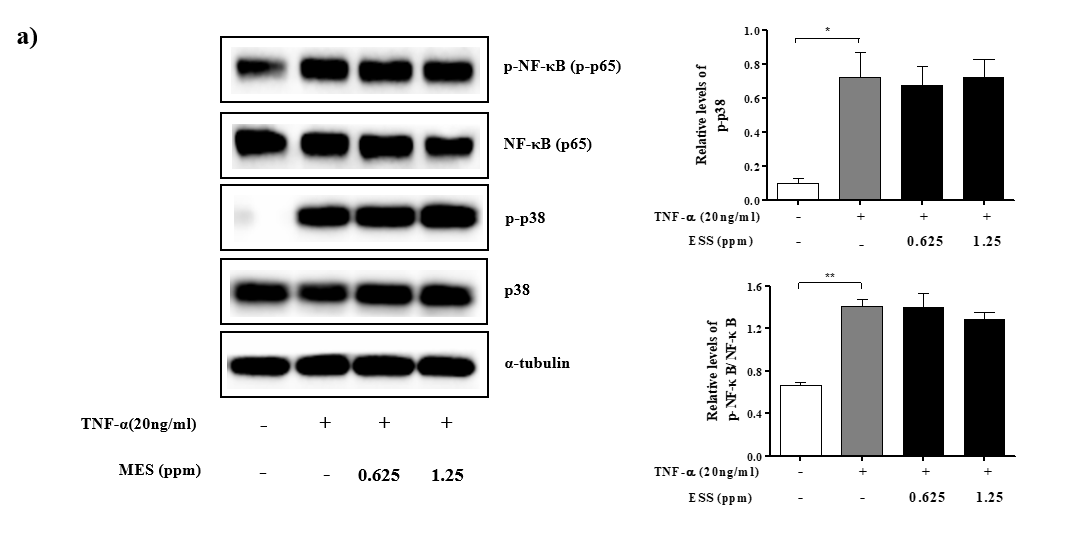
**

a) HaCaT cell were pretreated with different concentrations (0.625. 1.25 ppm) for 24 hours and then were stimulated with TNF-α for 30min-1hour. The phosphorylation of MAPKs was analyzed by western blot analysis. The phosphorylation of p38 and NF-κB was analyzed by the western blot. Phosphorylation of p38 and NF-κB was normalized by total form. Densitometric analysis was performed using a bioanalytical imaging system (Azure 300, AZI300-01, Azure biosystems, USA) and ImageJ (NIH, Rockville, MD, USA). The values presented are the mean ± SEM obtained from at least three independent experiments. Statistical significance is indicated as follows: ** p < 0.01, and * p < 0.05.


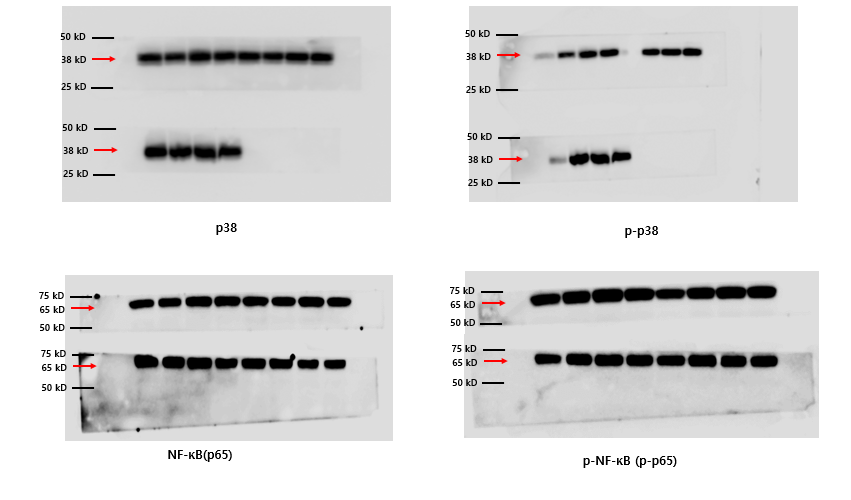


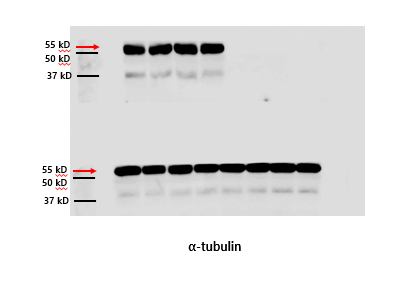


HaCaT cells (3×10^^5^ cells/well) were seeded onto a 6-well plate and cultured at 37°C for 48 hours. Following this, cells were pre-treated with ESS (0.625 and 1.25ppm) or DMSO for 1 hour, then stimulated with TNF-α (20ng/mL) for 30 minutes to 1 hour. After stimulation, cells were washed twice with cold PBS and protein extraction was performed on ice for 30 minutes using the ExKine™ Total Protein Extraction Kit (KTP3006, Abbkine, USA). The lysates were then centrifuged at 14,000 × g for 15 minutes, and the protein concentrations in the supernatants were measured. Equal amounts of protein (20 μg) were quantified and separated by SDS-polyacrylamide gel electrophoresis (PAGE), followed by transfer onto polyvinylidene difluoride (PVDF) membranes. The membranes were washed with Tris-buffered saline (TBS, 50 mM Tris-base, 200 mM NaCl, pH 7.4) containing 0.02% Tween 20 (TBST) and blocked with TBST containing 5% skim milk. Primary antibodies were added and incubated overnight at 4°C, followed by exposure to secondary antibodies for 1 hour and 30 minutes. The membranes were then washed three times with TBST at room temperature, and immunoreactivities were determined using an ECL detection kit. For multiplexing, the membrane was appropriately cut before primary antibody incubation to observe various proteins on a single membrane. Phospho proteins at the same position were probed again through stripping to enable band visualization. Densitometric analysis was carried out using the Azure 300 Chemiluminescent imaging system (AZI300-01, Azure biosystems, USA) and ImageJ (NIH, Rockville, MD, USA). It's noted that the Azure 300 Chemiluminescent imaging system automatically selects and saves the image with the clearest bands, potentially leading to obscured backgrounds or membrane edges.

**Figure S2. The mRNA expression for atopic dermatitis-related inflammatory markers in DNCB-induced HR-1 mice.**


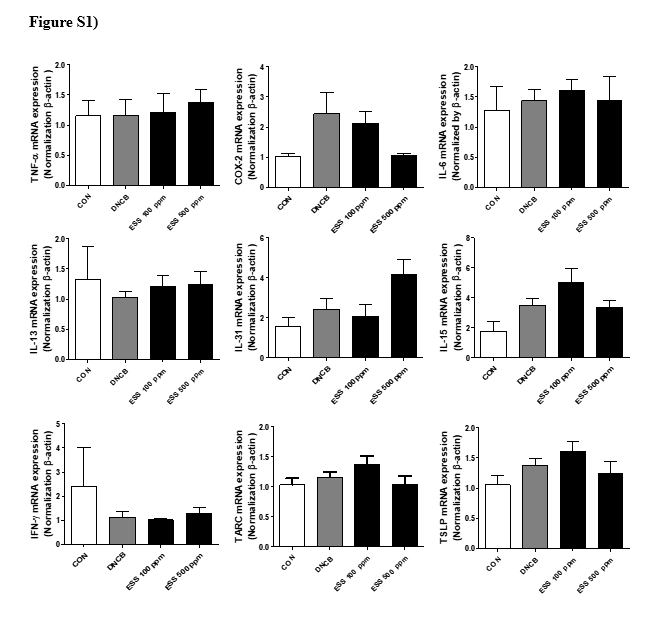


The modulation of inflammation-related gene expression in DNCB-induced HR-1 mouse skin by ESS treatment was investigated through RT-PCR. The values presented are the mean ± SEM obtained from at least seven independent experiments.
